# Varying conjunctival immune response adaptations of house finch populations to a rapidly evolving bacterial pathogen

**DOI:** 10.3389/fimmu.2024.1250818

**Published:** 2024-02-02

**Authors:** Nithya Kuttiyarthu Veetil, Amberleigh E. Henschen, Dana M. Hawley, Balraj Melepat, Rami A. Dalloul, Vladimír Beneš, James S. Adelman, Michal Vinkler

**Affiliations:** ^1^ Department of Zoology, Charles University, Faculty of Science, Prague, Czechia; ^2^ Department of Biological Sciences, The University of Memphis, Memphis, TN, United States; ^3^ Department of Biological Sciences, Virginia Tech, Blacksburg, VA, United States; ^4^ Department of Poultry Science, The University of Georgia, Athens, GA, United States; ^5^ European Molecular Biology Laboratory (EMBL), Genomics Core Facility, Heidelberg, Germany

**Keywords:** adaptations diversifying populations, emerging disease, coevolution, parasite, host-pathogen interaction, inflammatory immune response, resistance, tolerance to infection

## Abstract

Pathogen adaptations during host-pathogen co-evolution can cause the host balance between immunity and immunopathology to rapidly shift. However, little is known in natural disease systems about the immunological pathways optimised through the trade-off between immunity and self-damage. The evolutionary interaction between the conjunctival bacterial infection *Mycoplasma gallisepticum* (MG) and its avian host, the house finch (*Haemorhous mexicanus*), can provide insights into such adaptations in immune regulation. Here we use experimental infections to reveal immune variation in conjunctival tissue for house finches captured from four distinct populations differing in the length of their co-evolutionary histories with MG and their disease tolerance (defined as disease severity per pathogen load) in controlled infection studies. To differentiate contributions of host versus pathogen evolution, we compared house finch responses to one of two MG isolates: the original VA1994 isolate and a more evolutionarily derived one, VA2013. To identify differential gene expression involved in initiation of the immune response to MG, we performed 3’-end transcriptomic sequencing (QuantSeq) of samples from the infection site, conjunctiva, collected 3-days post-infection. In response to MG, we observed an increase in general pro-inflammatory signalling, as well as T-cell activation and IL17 pathway differentiation, associated with a decrease in the IL12/IL23 pathway signalling. The immune response was stronger in response to the evolutionarily derived MG isolate compared to the original one, consistent with known increases in MG virulence over time. The host populations differed namely in pre-activation immune gene expression, suggesting population-specific adaptations. Compared to other populations, finches from Virginia, which have the longest co-evolutionary history with MG, showed significantly higher expression of anti-inflammatory genes and Th1 mediators. This may explain the evolution of disease tolerance to MG infection in VA birds. We also show a potential modulating role of BCL10, a positive B- and T-cell regulator activating the NFKB signalling. Our results illuminate potential mechanisms of house finch adaptation to MG-induced immunopathology, contributing to understanding of the host evolutionary responses to pathogen-driven shifts in immunity-immunopathology trade-offs.

## Introduction

Host-parasite co-evolution belongs among the most dynamic evolutionary phenomena (1). Novel adaptations rapidly shift pathogen virulence [i.e. pathogen damage to host fitness (2)] as well as host immune defence capacities. Given the frequent emergence of novel zoonotic infections transmitted to humans from wildlife, there is urgent need for improved understanding of the natural variation in both patterns and mechanisms of host-pathogen evolution (3, 4). Despite common expectation that long-term coevolution between hosts and their pathogens favours decrease in the pathogen virulence (1), present evidence suggests variation in these evolutionary patterns, with long-term increase in virulence observed in certain contexts (5). In response, hosts can rapidly adjust their resistance, i.e. evolve capacity to decrease pathogen replication, consistent with the arms-race model (1). Such adaptations have emerged, for example, in amphibians (6) and bats (7) challenged by fungal pathogens, or rabbits facing myxoma virus epidemics (8). However, if pathology caused by the excessive immune defence is too costly (9), the immunity-immunopathology trade-off can favour the evolution of tolerance to the infection instead of, or in addition to, resistance (10–12). Unlike resistance, tolerance mitigates the host’s fitness loss through a reduction of tissue damage caused by infection or improved repair of this damage, without necessarily reducing pathogen replication. In contrast to resistance, evolution of tolerance to infection typically does not promote the arms race accelerating further increase in pathogen virulence (13, 14). However, if the increase in host’s tolerance decreases immunopathology that favours pathogen transmission, pathogen can respond by evolving higher virulence (15, 16). This can further select on optimisation of the immune response, setting equilibrium between host immunity and immunopathology (9). Although recent research in different species of wild vertebrates (17–19) indicated that infection tolerance can be a common strategy to reduce the fitness costs in hosts facing novel pathogens, we still mostly lack evidence on the immunological mechanisms responsible for the shifts between resistance to tolerance in natural host-pathogen systems.

One of the few relevant vertebrate models for this investigation where we have evidence for tolerogenic adaptation (20) can be found in the recent evolutionary interaction between the bacterium *Mycoplasma gallisepticum* (MG) and its novel host, the house finch (*Haemorhous mexicanus*) (21). MG is a horizontally transmitted pathogen that shows high antigenic variation (22). Previously known to be a respiratory pathogen of domestic poultry (23), in 1994 MG was first detected in wild house finches in Virginia (eastern USA), causing mild to severe conjunctivitis (24). Within three years, the infection spread across eastern North American populations of the host and, after a few-year’s lag, in the early 2000s the disease was detected in western North American house finch populations (25). Mycoplasmal conjunctivitis disease decreases survival of finches (26) in the wild, often causing severe decline (up to 60%) in affected house finch populations (27). However, the epizootic did not reach some isolated house finch populations, such as those introduced to the Hawaiian Islands which still remain naïve to MG. Further, because of the way that MG spread west across the northern part of the United States and then down the western coast, MG has only recently (or in some cases, never) been documented in host populations in areas of the southwest United States such as Arizona (28).

The house finch-MG model system is unique in avian evolutionary ecology given the precisely mapped spatiotemporal epizootic data and the wealth of pathogen isolates collected throughout time from various wild house finch populations that are presently available for infection experiments (29). This experimental research has shown that MG virulence has increased over time, with the evolutionarily original MG isolates (e.g. the isolate VA1994) causing milder disease than the more recent, evolutionarily derived isolates (e.g. the isolates NC2006 or VA2013) (30, 31). At the same time, there is inter-individual variability among hosts in their responses to the pathogen (32) and the host populations appear to have adapted to the MG selective pressure (33). We have recently shown that house finch populations with a longer co-evolutionary history with MG show more tolerance to the infection than the populations in recent or no contact with the pathogen (20), with tolerance quantified as milder disease severity (i.e., conjunctivitis) at a given pathogen load. This is probably linked to regulation of the inflammatory response, which is less pronounced in the Harderian glands of house finch populations in longer contact with the pathogen, compared with populations with little or no contact with MG (20, 33).

Bacteria of the genus *Mycoplasma* are extracellular and intracellular parasites known in vertebrates to trigger excessive proinflammatory signalling (e.g. mediated by *IL1B* or *IL6*), while down-regulating regulatory signals with anti-inflammatory effects (e.g. *IL10*) (34). In humans, clinical manifestations of acute mycoplasmosis result from immunopathologic inflammation generated by the host, rather than by the direct pathogen-mediated tissue damage (35). Excessive inflammation may contribute to MG’s ability to evade the host effector antibody response by disrupting regulation of the inflammation, improving pathogen transmission efficiency (36). In house finches, MG infection affects mainly the sites belonging to conjunctiva-associated lymphoid tissue, including conjunctiva and Harderian gland (37). Since its emergence in finches, MG appears to have evolved to trigger stronger pro-inflammatory cytokine levels in the host periocular lymphoid tissues, which is positively correlated with increased bacterial loads (37), disease severity (5), and pathogen spreadability (36). This promotes in the host an evolutionary trade-off between selection on stronger immunity to clear the pathogen infection, consistent with resistance, and constraint emerging from immunopathology, selecting on down-regulation of inflammation achieved through tolerance.

Transcriptomic analysis is an important approach to identify possible shifts in immune regulation of host-pathogen interactions. Previous studies using transcriptomics in house finches focused on gene expression changes in spleen, a secondary lymphoid tissue not topologically linked with the MG infection site where the primary direct contact between the host and the pathogen occurs (38, 39). Our previous RNA-seq transcriptomic research in the Harderian gland (20), a periocular secondary lymphoid tissue, has shown that 3 days post inoculation (DPI) with MG, house finches from more tolerant populations (those with a longer history of MG endemism) also showed reduced up-regulation of immune gene expression, notably among inflammation-regulating chemokines (20). Here we adopted the 3’-end transcriptomic QuantSeq approach to more closely explore the variation in immune regulation underlying the observed differences between the house finch populations in their tolerance to MG. Unlike the previously studied Harderian gland, conjunctiva is a lymphoid tissue directly exposed to the MG pathogen and thus the first tissue to be immunologically affected by the infection. Our objective was to describe the conjunctival immune response involved in directing the subsequent pathway regulation towards resistance or tolerance to MG. We used samples from the same birds for which Harderian gland tissues were analysed in Henschen et al. (20). MG-naïve house finch juveniles that were captured in one of four wild populations (Virginia = VA, Iowa = IA, Arizona = AZ and Hawaii = HI) were exposed to one of two MG isolates (original VA1994 or evolved VA2013) under controlled captive conditions. At the time of experimentation, the VA population had experienced the longest coevolution with MG (>20 years), the IA population only a slightly shorter co-evolution with MG than VA (~20 years (24);, while in AZ the MG epidemics are still relatively recent (0-5 years, with no detections in the population sampled (28);, and the HI population is likely entirely naïve to MG due to its geographic isolation (20). Differences between house finch populations in their co-evolutionary time with MG allowed us to track the variation in the immune responses associated with adaptation to the pathogen. The immune responses were assessed 3 DPI in order to describe the initial phase of the infection, during which innate immune regulation is being established at the infection site (37). Using differential gene expression (DGE) analysis, we first identified the immune pathways involved in response to MG and their differences between the four host populations (model 1). In our analysis, we focused namely on the variation in pro-inflammatory pathways that could promote resistance to MG and regulatory mechanisms that could increase tolerance to MG, indicating house finch adaptations to the pathogen. Second, we described differences between the four host populations in control individuals, where variation in baseline immune regulation can be identified (model 2). Third, we characterised differences in conjunctival immune responses associated with MG strain virulence (model 3).

## Materials and methods

### Experimental design and animals

Details of the experiment are provided in (20), so here we recapitulate it only briefly. Hatch-year house finches (identified as first-year based on plumage characteristics) were captured using mist nets and feeder traps (40) between June and September 2018 in Blacksburg, Virginia (VA), Ames, Iowa (IA), Tempe, Arizona (AZ) and Oahu, Hawaii (HI) (details provided in **Supplementary Table S1**, Electronic **Supplementary Material 1**, ESM1 and map displaying the details of sample collection is shown in **Supplementary Figure 1**, **Supplementary Figure S1** in ESM2). Any finches that showed clinical signs of MG infection during capture were immediately released. Following capture, each bird received a uniquely numbered aluminium leg band, and an electronic balance was used to determine its mass. To eliminate ectoparasites, the birds were all dusted with 5% sevin powder. The trapped birds were brought to the Iowa State University animal facility. After arrival, all birds were subjected to an acclimation and quarantine period (minimum of 40 days), which included treatment with prophylactic medications to prevent naturally occurring infections. A serological assay was run on blood collected approximately two weeks post-capture to ensure that all birds used in experiments were seronegative for MG infection (20).

Birds were kept individually in medium flight cages (76 cm x 46 cm x 46 cm) for the duration of the experiment and were provided *ad libitum* access to water and food. The diet consisted of a 20:80 mixture of black oil sunflower seeds and pellets (Roudybush Maintenance Nibles; Roudybush, Inc., Woodland, CA). Temperatures (~22°C) and light-dark cycles (12h:12h) were kept constant.

The infection experiment was performed in October 2018 on a sample of 60 individuals representing the four different house finch populations (VA, IA, AZ, HI). For each population, 5 individuals served as controls (C) treated with Frey’s media with 15% swine serum alone, 5 were treatment individuals inoculated with the original MG isolate VA1994, and 5 were inoculated with the evolved MG isolate VA2013 (in both treatments the MG dose was 7.5×10^6^ colour changing units, CCU/mL) following the same methodology as in (5, 41). Three days post-infection (3 DPI), the birds were euthanised by rapid decapitation and a panel of nine tissues were collected. All tissues were submerged into RNA later protectant within 15 minutes of euthanasia and immediately refrigerated at 4°C. The cooled periocular conjunctiva-associated lymphoid tissue (conjunctiva and nictitating membrane) samples were transported within 48 hours to Charles University, Czech Republic, where they were kept frozen to -80°C until further processing.

### RNA extraction and sequencing

Our conjunctival samples contained both the conjunctiva-associated lymphoid tissue (CALT) and skin of the eye lid. For ensuring the proper RNA extraction of the lymphoid tissue, we used the following protocol. All conjunctival samples from the 60 birds were homogenized using PCR-clean beaded tubes (OMNI International, USA - Serial Number: 2150600) using the MagNa Lyser (Roche, Basel, Switzerland). The skin tissues present in the samples were separated during the centrifugation step and discarded, while the homogenised lymphoid tissue was used for the total RNA extraction with the High Pure RNA Tissue Kit (Roche, Basel, Switzerland). We used Nanodrop (NanoDrop ND-1000) and Agilent 2100 Bioanalyzer with nano chip (Agilent Technologies, California, USA) to calculate the RNA yield (in all cases >20 ng/ul) and integrity (in all cases RIN values >7) (details provided in **Supplementary Table S2**, ESM1).

To perform sufficiently deep transcriptomic sequencing in a representative sample of individuals with different treatments across four populations, we adopted the 3’-end transcriptomic QuantSeq approach, which is more cost-efficient in larger population samples than the classical RNA-seq ( (42–44); Kuttiyarthu Veetil et al. in prep.). The library preparation and sequencing were performed at the European Molecular Biology Laboratory (EMBL), Heidelberg, Germany. All the samples were first barcoded with Illumina TruSeq adapters (45). The QuantSeq libraries were prepared using Lexogen QuantSeq 3’-polyadenylated RNA Library Prep Kit FWD (Illumina). The sequencing was carried out using the Illumina NextSeq 500 platform. QuantSeq is based on a protocol devoid of mRNAs fragmentation before reverse transcription (46), but the read fragment sequencing targets are generated close to the polyadenylated 3′ end. This method uses total RNA as an input and there is no prior poly(A) enrichment or rRNA depletion. QuantSeq generates only one read fragment per transcript, and the number of reads mapped to a given gene is, therefore, proportional to its expression (42). Eight samples failed during library preparation and were excluded from the sequencing. The rest of the 52 indexed samples were pooled together and single-end 80 bp reads were generated. Thus, the final analysis is based on the sequence data representing conjunctival samples from 52 birds (details on the birds provided in **Supplementary Table S3**, ESM1).

### Transcriptomes

On average, we obtained ~10 million reads per sample, comparable to zebra finch 3’-end transcriptomic sequencing. The bioinformatic analysis was carried out using BAQCOM pipeline (https://github.com/hanielcedraz/BAQCOM). The samples were aligned to the zebra finch genome downloaded from Ensembl (47) (bTaeGut1_v1.p-GCA_003957565.1). The tools included Trimmomatic (version 0.39) (48) for the adapter trimming, STAR software (49) for the aligning with the reference and feature Counts from the Subread package (50) for assigning of the sequences and gene level quantification. The alignment percentage of the conjunctiva samples to the reference genome ranged between 52.42% to 80.62% (**Supplementary Table S4**, ESM1). Next, the DGE analysis was performed using the limma (Linear Models for Microarray Data) package (51) in R (version- version 4.1.1) (52). In this analysis, we considered the source population, sex, and MG treatment as fixed factors, testing them together with their interactions at the significance level of padj value ≤ 0.05 and a minimum log2fold change value ≥1. After the differential gene expression analysis, each gene in each transcriptome was annotated. Ensembl BioMart (47) was used to assign gene functional annotations (geneontology, GO), which were then manually supplemented with Uniprot annotations. In cases where gene names were not directly available, an orthologue search was performed (Ensembl and NCBI Blast) for human annotations and gene names were selected if the closest hit showed at least 60% sequence identity. We used ShinyGO (version-0.77) (53) for generating the figures for pathway analysis and using Venn (https://bioinformatics.psb.ugent.be/webtools/Venn/) to create the venn diagrams. The transcriptomic sequenced data were submitted to the NCBI Sequence Read Archive. As an alternative, guided by our research question, literature search (54) and previous results (33), we selected the following target cytokine and receptor genes potentially involved in regulation of the house finch immune interaction with MG: *IL1B, IL10, IL6, CXCL8, IL22, TNFSF15, TLR4, TLR3, TLR2, ACOD1*, *CSF1R, CCL4, IL18*, and *TLR7* (selected based on literature search and 3’ end annotation availability; **Supplementary Table S11**, ESM1).

### Statistical analysis

To identify potential transcriptomic groupings of our four populations, we first performed two Between group analyses (BGA) using made4 package in R (55). In the first analysis, we used the individual population identities as a grouping factor, while for the second analysis we adopted the distinction between eastern populations (VA and IA), which share a long co-evolutionary history with MG, and western (AZ and HI) populations which share a short (0-5 year) co-evolutionary history with MG, as applied in our previous research (20). BGA targets the between-group variability by executing a principal component analysis (PCA) on group means.

Next, we adopted three different methodological strategies to reveal the transcriptomic variation between the house finch populations and the two MG isolates using limma package from R. Limma employs moderated t-statistics to assess differences in expression of individual genes across the transcriptome. It allows to design multiple-factor matrices (e.g., different time points, experimental conditions, batch effects) and covariates, from which it calculates the differential gene expression by accounting for all the variables. Limma generates a full list of genes with associated p-values and false discovery rate (FDR) for each gene, indicating the result reliability (51).

First, to reveal population-specific variation in immune responses to MG among the four house finch populations, in the whole dataset we tested the following linear model, considering population of origin, sex, MG treatment and the interaction between population and MG treatment as explanatory variables (model 1):


(∼ Population + Sex + MG_treatment + Population: MG_treatment + MG_treatment: Sex)

The target-gene analysis was performed only using the whole dataset. To normalize the target gene expression data, we first divided the total number of reference-aligned reads by the total number of reads in the sample (Cn). To scale the data, we then multiplied each of the normalized read counts by 10 million (approx. 10 million was the average number of reads per sample in our dataset). Given large number of zero expression levels detected, we could not make relative quantification of the expression and, therefore, the variation in gene expression is shown as a logarithm of the scaled-normalized read counts, with uniform scaling across all genes. These gene expression levels were visualised using heatmap: pheatmap package in R.

Since the results of model 1 indicated limited Population : MG_treatment interactions, but revealed main effects of the populations, to understand the pre-existing variation in gene expression among those populations we then run a second linear model, where in the control individuals alone we tested the parameters of population, sex and their interaction (model 2):


(∼ Population + Sex + Population: Sex)

Third, to reveal the differences in immunity activation caused by the two MG isolates used (the original VA1994 vs. evolved VA2013), we finally separately analysed the DGE in the VA2013 treatments compared to the controls, and in the VA1994 treatments compared to the controls, later contrasting the two sets of results (model 3):


(∼ Population + MG_treatment + Population: MG_treatment)

## Results

**Figure 1:**
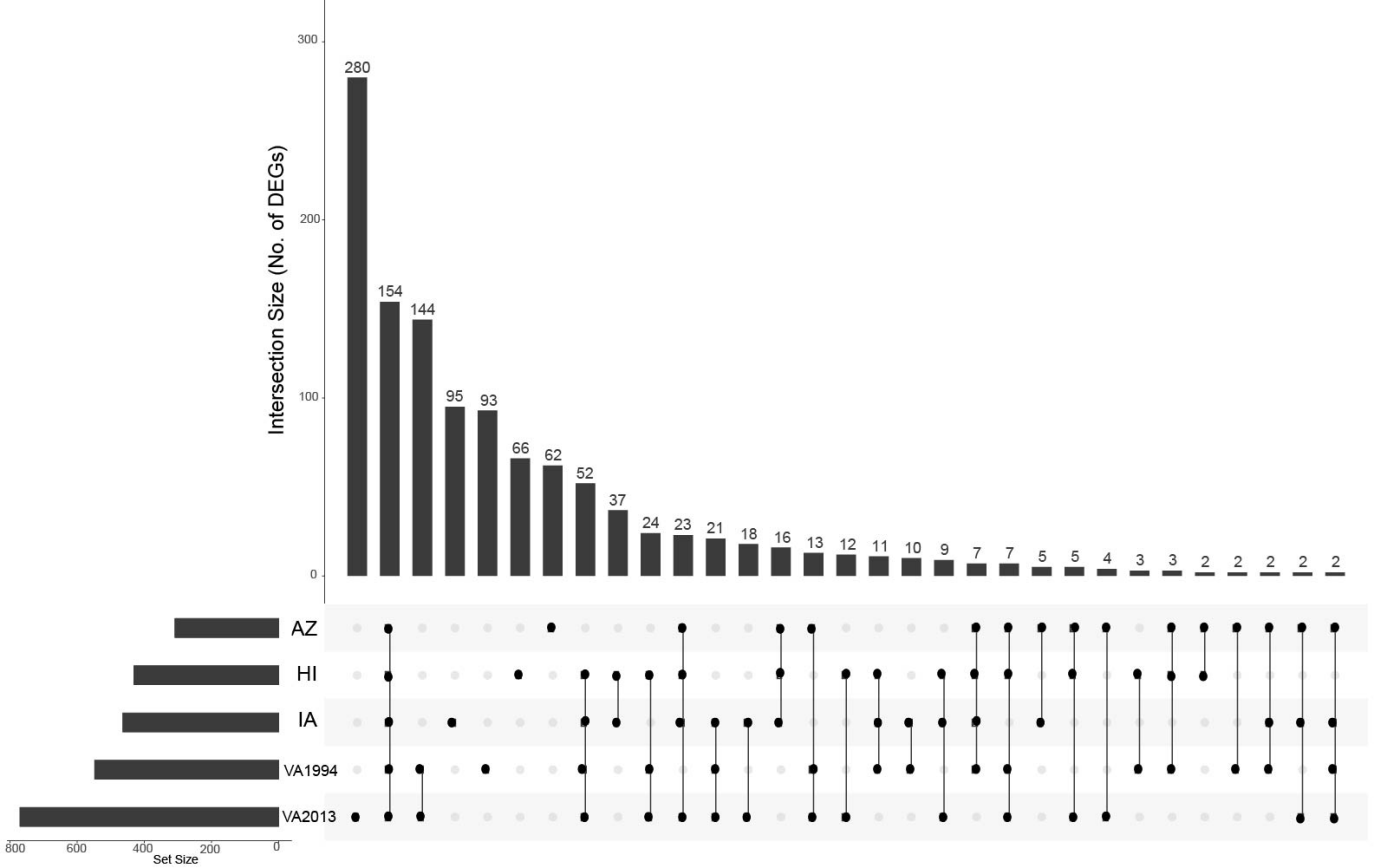
UpSet plot depicting the common differentially expressed genes in conjunctival tissue across the investigated house finch populations and the *Mycoplasma gallisepticum* (MG) treatments. The house finch populations namely, Arizona (AZ), Iowa (IA) and Hawaii (HI) are compared with the Virginia (VA) population, the MG treatments (VA1994 and VA2013) are compared with the controls. The gene set size is represented by the bar height, and the population-treatment interaction by the lines connecting the main category dots.

First, to identify general transcriptomic similarities between birds from different populations, we performed the between-group analyses (BGA) comparing individual populations and their western and eastern sets. These did not reveal any clear grouping of the individuals based on their transcriptomic profiles (P>0.05; **Supplementary Figures S2**, **S3**, ESM2). To investigate variation among house finch populations in their responsiveness to MG infection, we first performed a general analysis on the whole dataset (model 1). In total we identified 1228 DEGs (**Figure 1**; **Table 1**; heatmap is provided in **Supplementary Figure S4**, ESM2). Among the 23 genes which were differentially expressed between sexes, none showed any interaction with the MG treatment, and none were involved in immunity, indicating no sex-specific variation in immune responses to MG in the conjunctival gene expression. Therefore, sex effects were not further considered in our analysis.

**Table 1:** Results of the general differential gene expression (DGE) analysis for conjunctival tissue collected 3 days post inoculation with *Mycoplasma gallisepticum* (MG) treatment (model 1).

Factors	Total DEG	Total Up	Total Down	Immune DEG	Immune Up	Immune Down
**AZ**	309	141	168	17	15	2
**HI**	431	151	280	29	24	5
**IA**	464	131	333	18	15	3
**VA1994**	548	310	238	76	71	5
**VA2013**	772	444	328	91	81	10
**AZ : VA1994**	5	0	5	0	0	0
**AZ : VA2013**	1	0	1	0	0	0
**HI : VA1994**	6	2	4	2	0	2
**HI : VA2013**	2	0	2	1	0	1
**IA : VA1994**	1	0	1	1	0	1
**IA : VA2013**	0	0	0	0	0	0
**SEX**	23	15	8	0	0	0
**VA1994:SEX**	0	0	0	0	0	0
**VA2013:SEX**	0	0	0	0	0	0

The table shows the total numbers of differentially expressed genes (Total DEG) and the total numbers of differentially expressed immune genes (Immune DEG) across different comparisons as well as numbers of up-regulated (Up) and down-regulated (Down) genes for the two infection treatments (VA1994 and VA2013) compared to controls and the populations Arizona (AZ), Hawaii (HI) and Iowa (IA) when compared to the Virginia (VA) population, including interactions.

Regardless of the MG treatment status, compared to the VA population, most DEGs were observed in the IA population (464), indicating baseline differences between these two populations in conjunctival gene expression. Though high number of DEGs were detected between both the MG treatments and controls (548 for VA1994 and 772 for VA2013), there was little interaction between MG treatment and house finch population origin (**Table 1**). To indicate the overlaps between the populations and MG treatments, we provide the UpSet plot in **Figure 1**. Among the 154 genes on the overlap of all groups, the majority of the genes were lacking any annotations (representing novel transcripts) and there were no genes annotated with any immune function.

**Figure 2:**
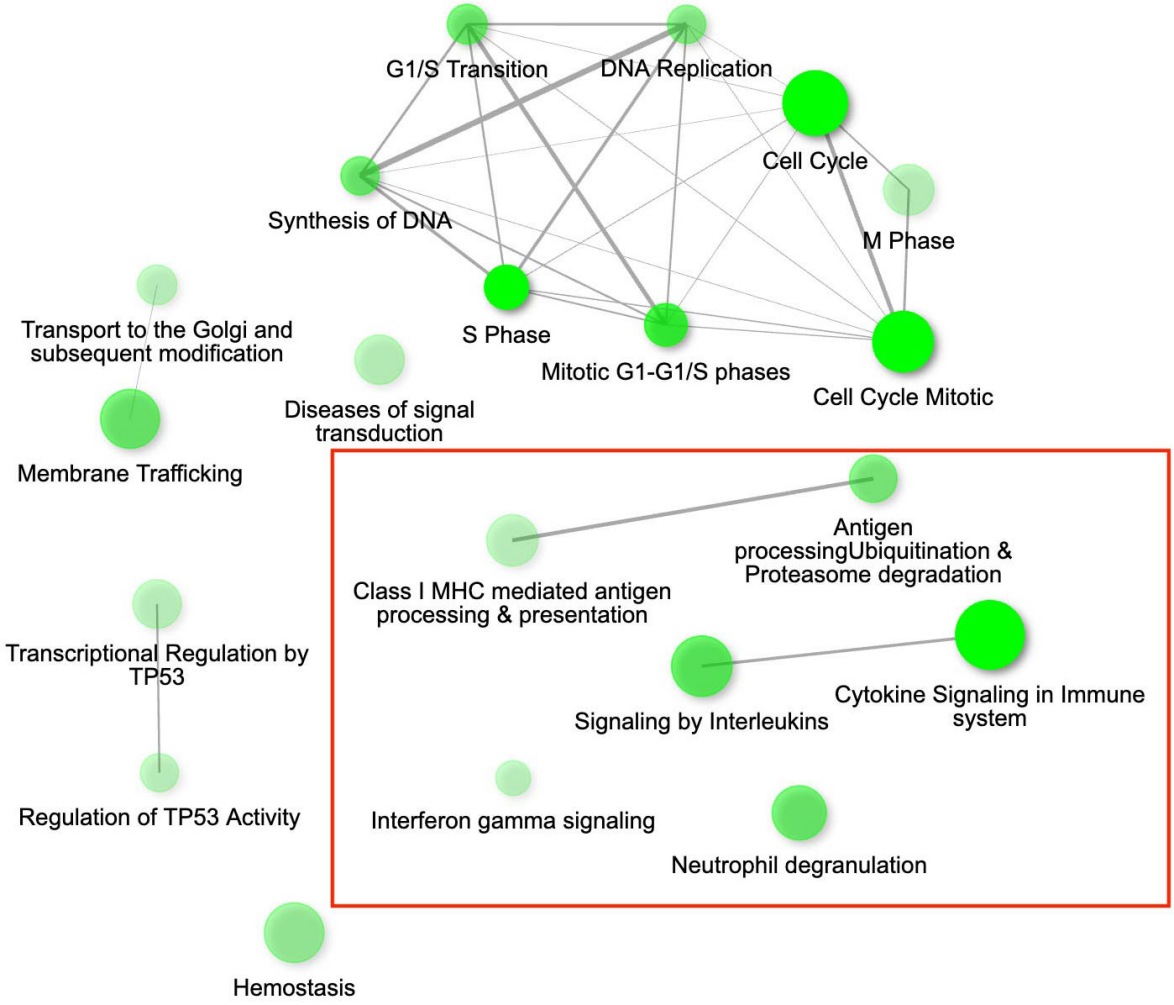
The gene interaction network for the differentially expressed genes (DEGs) up-regulated in conjunctival tissue 3 days post inoculation (DPI) with *Mycoplasma gallisepticum* (infected vs. non-infected birds across all house finch populations), showing the most significant pathways in the GO category Biological process. Immune genes grouped in the pathways of our interest are highlighted with red rectangles. Node colour intensity indicates significance of gene enrichment, node size indicates number of significant DEGs.

While we identified in total 900 DEGs related to MG infection (across all population, combining VA1994 and VA2013, with the main effects and interactions), only 793 were annotated (**Supplementary Table S5**, ESM1), and among those we identified 113 DEGs involved in immunity (**Supplementary Table S6**, ESM1). There were 158 annotated DEGs down-regulated in their expression during MG infection. For example, *CHRNB2*, *ATP2B1*, *SCN2A*, *RYR2*, *NKAIN1* and *CACNA1C* are important for the ion transport [GO:0006811], synaptic signalling [GO:0032225] and response to muscle activity [GO:0014850] (**Supplementary Figure S5**, ESM2). Only 11 out of the 158 down-regulated genes showed clear links to immunity, including *IL12B* and *RAG1* that are involved in Th1/Th17 immune response activation [GO:0032735, GO:0032740], positive regulation of T cell differentiation [GO:0045582], pre-B cell allelic exclusion [GO:0002331] and adaptive immune response [GO:0002250]. Among the 457 annotated DEGs up-regulated during MG infection, we were able to identify 91 genes with immune function. In the MG-treated individuals, we observed increased expression of, e.g. *IL17RA* and *IL17RE* involved in inflammatory response [GO:0050729], regulation through IL17-mediated signalling pathway [GO:0097400], *CXCL12* involved in defence response [GO:0006952], *TLR1B* activating toll-like receptor TLR6:TLR2 signaling pathway [GO:0038124], a leukocyte marker *PTPRC*(*CD45*) regulating T cell proliferation [GO:0042102], *ACOD1* involved in positive regulation of antimicrobial humoral response [GO:0002760] and negative regulation of the inflammatory responses (56), and *CD74* involved in antigen processing and presentation [GO:0019882]. The main pathways in which the genes were up-regulated during MG infection are shown in **Figure 2**. Interestingly, while not statistically significant, *IL22* gene that plays a critical role in modulating tissue responses during inflammation [GO:0005125, GO:0006954], was found to be close to significance with increased expression in the birds treated with the VA2013 isolate (padj cut-off value = 0.07).

There were few genes for which we detected significant interactions between population and MG treatment (**Supplementary Table S7**, ESM1). Out of these, only 3 genes were involved in immune regulation. *BCL10* (positive regulation of interleukin-6 production [GO:0032755]; positive regulation of interleukin-8 production [GO:0032757], positive regulation of NFKB transcription factor activity [GO:0051092]; having roles in both innate immune response [GO:0045087] and adaptive immune response [GO:0002250]) was significantly differentially expressed in interaction between both HI and IA population and treatment with the MG isolate VA1994. During MG infection, *BCL10* was down-regulated in these populations. *CNN2* (actomyosin structure organization [GO:0031032]) and *TRIM13* (innate immune response [GO:0045087]; positive regulation of cell death [GO:0010942]) were detected differentially expressed in interaction between HI population and VA1994.

In the same analysis, a large number of DEGs were revealed between different house finch populations, regardless of the MG infection. In AZ birds, out of the 309 DEGs identified (**Supplementary Table S8**, ESM1) we were able to annotate 106 genes with expression higher and 35 genes with expression lower than in the VA population. There were 17 genes with immune-related functions, out of which 15 genes showed higher expression in AZ than in VA, including e.g., *BCL10*, *IL17D* involved in positive regulation of interleukin-8 production [GO:0032757] and *CASP6* involved in activation of innate immune response [GO:0002218]. The main immune gene with lower expression in AZ versus VA birds was *NR1H4* involved in negative regulation of IL1 [GO:0032692] production and inflammatory response [GO:0050728]. For HI birds, we found 431 DEGs, out of which 130 annotated genes had higher and 81 genes lower expression than in the VA population (**Supplementary Table S9**, ESM1). There were 28 genes linked with immune functions, again most of them (23 genes) having higher expression in HI than in the VA population. Like in AZ, these genes included *BCL10* and *CASP6*, but also *MAST2* involved in negative regulation of IL12 production [GO:0032655]. The immune genes with lower expression in HI relative to VA were *NR1H4*, *RAG1* and *KPNA6* involved in positive regulation of cytokine production involved in inflammatory response [GO:1900017]. In the IA population we found as many as 464 DEGs compared to the VA population (**Supplementary Table S10**, ESM1), among which 114 annotated genes showed higher expression and 80 genes lower expression than in the VA population. Among the 17 genes annotated with immune function, 15 (including again *BCL10* and *CASP6*, and *TRIM13*) had higher expression and two genes (*RAG1* and *NR1H4*) lower expression in IA than in VA. Thus, our results indicate that there is important variation between the house finch populations in immune gene expression in conjunctival tissue that is independent of the actual MG treatment (no significant effect of the interaction between the MG treatment and population).

**Figure 3:**
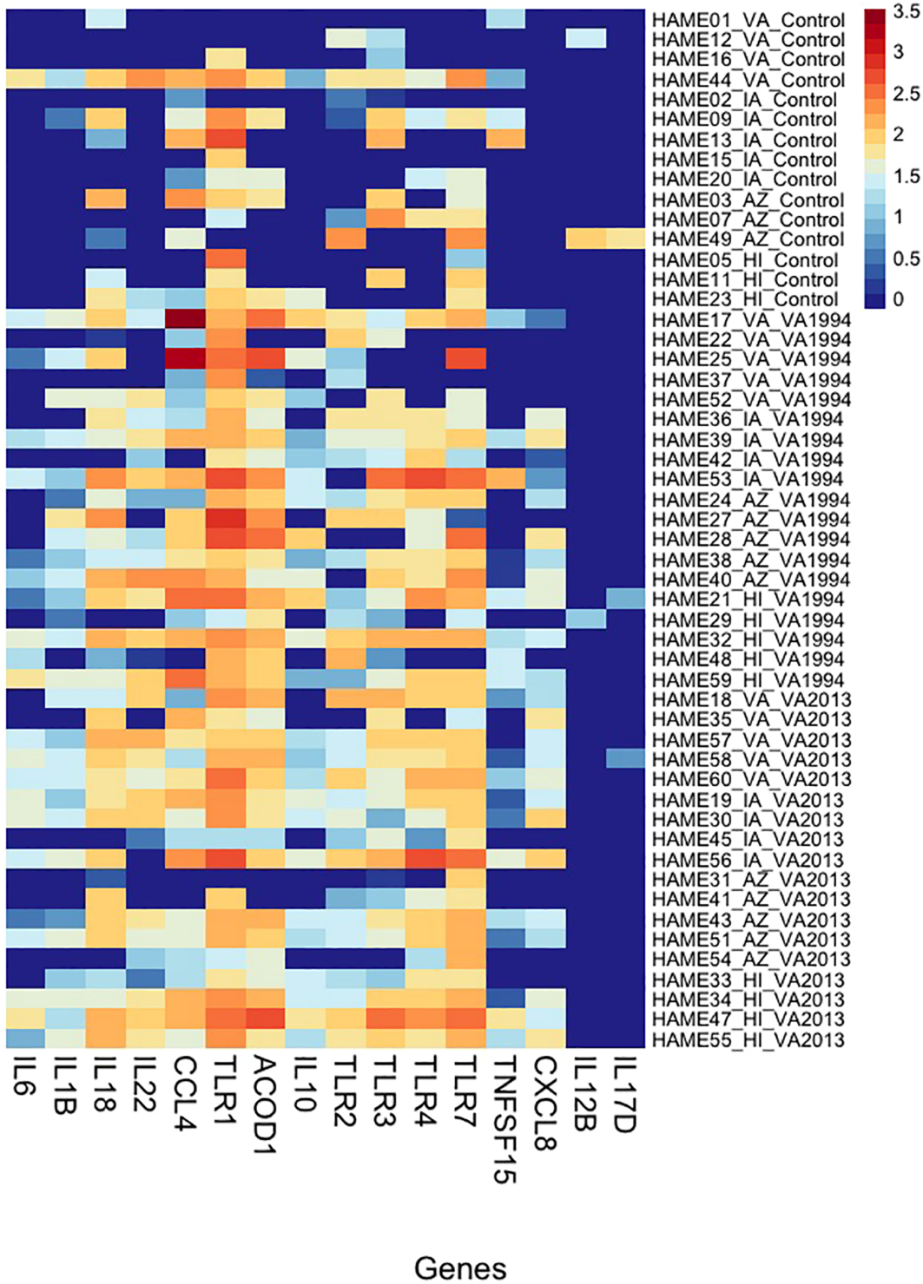
Heatmap showing variation in gene expression in selected inflammation-regulating genes (cytokines and receptors) in conjunctiva across house finches from four different populations belonging to two types of *Mycoplasma gallisepticum* (MG)-infected treatments (VA1994 and VA2013) and controls. Y-axis provides the information on individual birds (including population name and treatment group); X-axis shows the gene names; colour indicates the gene expression levels shown as a logarithm of the scaled-normalized read count varying from low expression (dark blue) to high expression (red). Please note that the scaling is not relative and, therefore, the colour pattern is common to all genes (highly as well as lowly expressed).

As an alternative approach, we also checked for the relative DGE changes in selected key immune genes with regulatory roles in immunity (target-gene analysis; **Supplementary Table S11**, ESM1) between the control and treatment groups of birds from different populations. Our results (statistics provided in **Supplementary Table S12**, ESM1) find that *IL1B, IL6*, *IL10*, *IL12B*, *IL17D*, *IL18*, *IL22*, *CXCL8*, *CCL4*, *ACOD1*, *TLR1*, *TLR4* and *TLR7* show clear distinction between the controls and the MG treatment groups (**Figure 3**), and at the same time *CCL4*, *TLR1*, *TLR4*, *TLR7* show also significant variation in expression between the populations. In *TLR1*, we even detected significant interaction between the MG treatment and population (AZ, HI) indicating differences in DGE between the populations in response to MG infection.

### Immune genes differentially expressed between populations in the unstimulated controls

Since the differences between the house finch populations in expression of immune genes were largely independent of MG infection status, indicating potential population-specific adaptations to MG, we also checked for differences in immune regulation in the unstimulated control individuals across populations (model 2). Our analysis showed 748 DEGs in the control individuals, with 71 genes (out of the 498 genes with defined annotations) being involved in immunity (**Table 2**).

**Table 2:** Results of the general differential gene expression (DGE) analysis in conjunctival tissue of control individuals (model 2).

Factors	Total DEG	Total Up	Total Down	Immune DEG	Immune Up	Immune Down
**AZ**	342	152	190	40	18	22
**HI**	270	55	215	31	8	23
**IA**	281	63	218	39	11	28

The table shows the total numbers of differentially expressed genes (Total DEG) and the total numbers of differentially expressed immune genes (Immune DEG) across the Arizona (AZ), Iowa (IA) and Hawaii (HI) and Virginia (VA) populations. Up = up-regulated (increased expression) in the tested population compared to VA, Down = down-regulated (decreased expression) in the tested population compared to the VA population.

**Figure 4:**
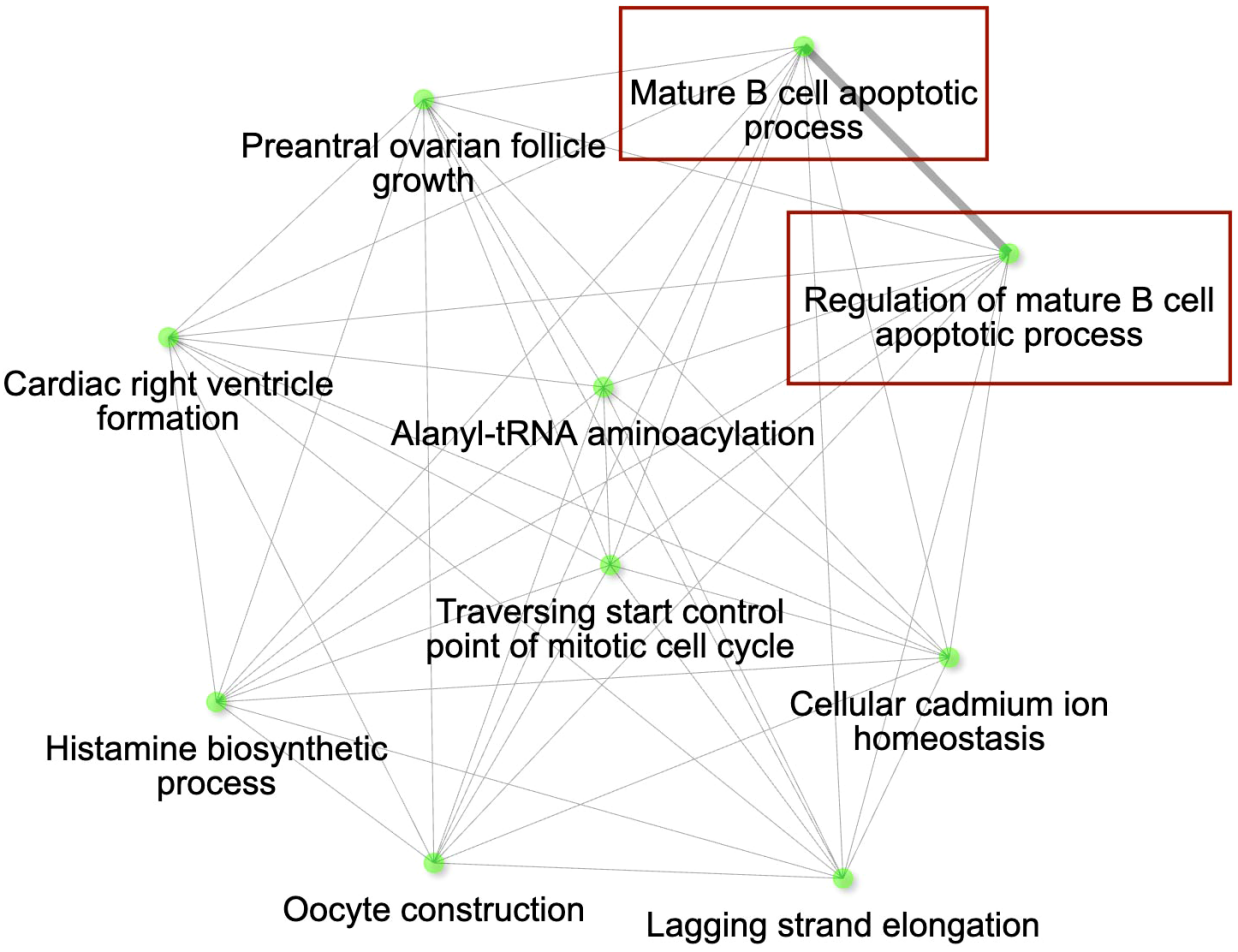
The gene interaction network for the differentially expressed genes (DEGs) with higher expression in conjunctiva of control birds in Iowa (IA), Arizona (AZ) and Hawaii (HI) compared to Virginia (VA). The most significant pathways in the GO category Biological process are shown. Immune genes grouped in the pathways of our interest are highlighted with red rectangles. Node colour intensity indicates significance of gene enrichment, node size indicates number of significant DEGs.

The lists of genes with lower expression in AZ, IA and HI populations compared to the VA population (**Supplementary Table S13**, ESM1) were mostly consistent (**Supplementary Figure S6**, ESM2), indicating generally increased expression of the genes in the VA birds: out of the 31 DEGs with immune function, 19 were shared between AZ, IA and HI birds. Notably, these included *LIF* (having role in regulation of immune response [GO:0050776] and anti-inflammatory properties; (57)], *IL12B* and *IL7* [positive regulation of T cell differentiation [GO:0045582] and cytokine-mediated signaling pathway [GO:0001961]). Among the 184 genes (**Supplementary Table S14**, ESM1) that were consistently expressed at higher levels in other populations compared to VA, 35 genes (**Supplementary Table S15**, ESM1) were shared between the AZ, HI and IA, indicating decreased expression in the VA population. There were 25 DEGs annotated with immune function which had higher expression across these three populations when compared to VA birds. Out of them, however, only 4 genes were shared: *BCL10*, *GGT5* (role in inflammatory response [GO:0006954]), *RABGEF1* (negative regulation of inflammatory response [GO:0050728]) and *SYNCRIP* (cellular response to interferon-gamma [GO:0071346]) (**Figure 4**).

The main uniquely up-regulated immune genes (18 genes) in the AZ population included *IL17D, IL17C* (inflammatory response [GO:0006954])*, IRF6* (immune system process [GO:0002376]), *TLR15* (toll-like receptor signaling pathway [GO:0002224]) and *TLR1B* genes (up-regulated and down-regulated pathways are shown in **Supplementary Figures S7**, **S8**, ESM2). In contrast to AZ, the HI and IA populations (up-regulated and down-regulated pathways for IA and HI birds, respectively, are shown in **Supplementary Figures S9**–**S12**, ESM2) showed almost identical sets of DEGs in the control birds: out of a total of 40 DEGs with immune function revealed in these populations, 28 genes were shared between these two populations, including *TRIM13, PPARD* (negative regulation of inflammatory response [GO:0050728]) and *BCAR1* (antigen receptor-mediated signaling pathway [GO:0050851]) that were different from the AZ population. These genes are involved in immune pathways involved in cytokine production by mast cells and B cells.

### Immune genes differentially expressed between individuals inoculated with different MG isolates

Our third analysis (model 3) showed only 160 DEGs for the MG VA1994 isolate, but 1229 DEGs for the VA2013 isolate (**Table 3**). Considering only the genes with annotations related to immune function, there were 54 genes differentially expressed during the infection with VA1994 and 230 genes during the infection with VA2013. In birds infected with VA1994, all the differentially expressed immune genes showed higher expression when compared to control birds. In birds infected with VA2013, there were 191 genes with higher expression and 39 genes with lower expression when compared to the controls (full list of the genes is provided in **Supplementary Tables S16**, **S17**, ESM1).

**Table 3:** Results of the differential gene expression (DGE) analysis in conjunctival tissue collected 3 days post inoculation with VA1994 and VA2013 isolates of *Mycoplasma gallisepticum* (MG) analysed separately (model 3).

Factors	Total DEG	Total Up	Total Down	Immune DEG	Immune Up	Immune Down
**VA1994**	160	148	12	22	22	0
**AZ**	6	6	0	0	0	0
**HI**	2	2	0	0	0	0
**IA**	14	11	3	0	1	0
**VA1994:AZ**	0	0	0	0	0	0
**VA1994:HI**	0	0	0	0	0	0
**VA1994:IA**	0	0	0	0	0	0
**VA2013**	1229	785	444	178	139	39
**AZ**	34	26	8	3	3	0
**HI**	45	28	17	3	2	1
**IA**	47	37	10	2	2	0
**VA2013:AZ**	2	0	2	0	0	0
**VA2013:HI**	2	1	1	0	0	0
**VA2013:IA**	1	1	0	0	0	0

The table shows the total numbers of differentially expressed genes (Total DEG) and the total numbers of differentially expressed immune genes (Immune DEG) for the MG isolates (Va1994 and VA2013), populations (AZ, Arizona; HI, Hawaii; IA, Iowa; VA, Virginia) and their interactions. Up = up-regulated compared to controls/increased expression in the tested population compared to VA, Down = down-regulated compared to controls/decreased expression in the tested population compared to the VA population.

**Figure 5:**
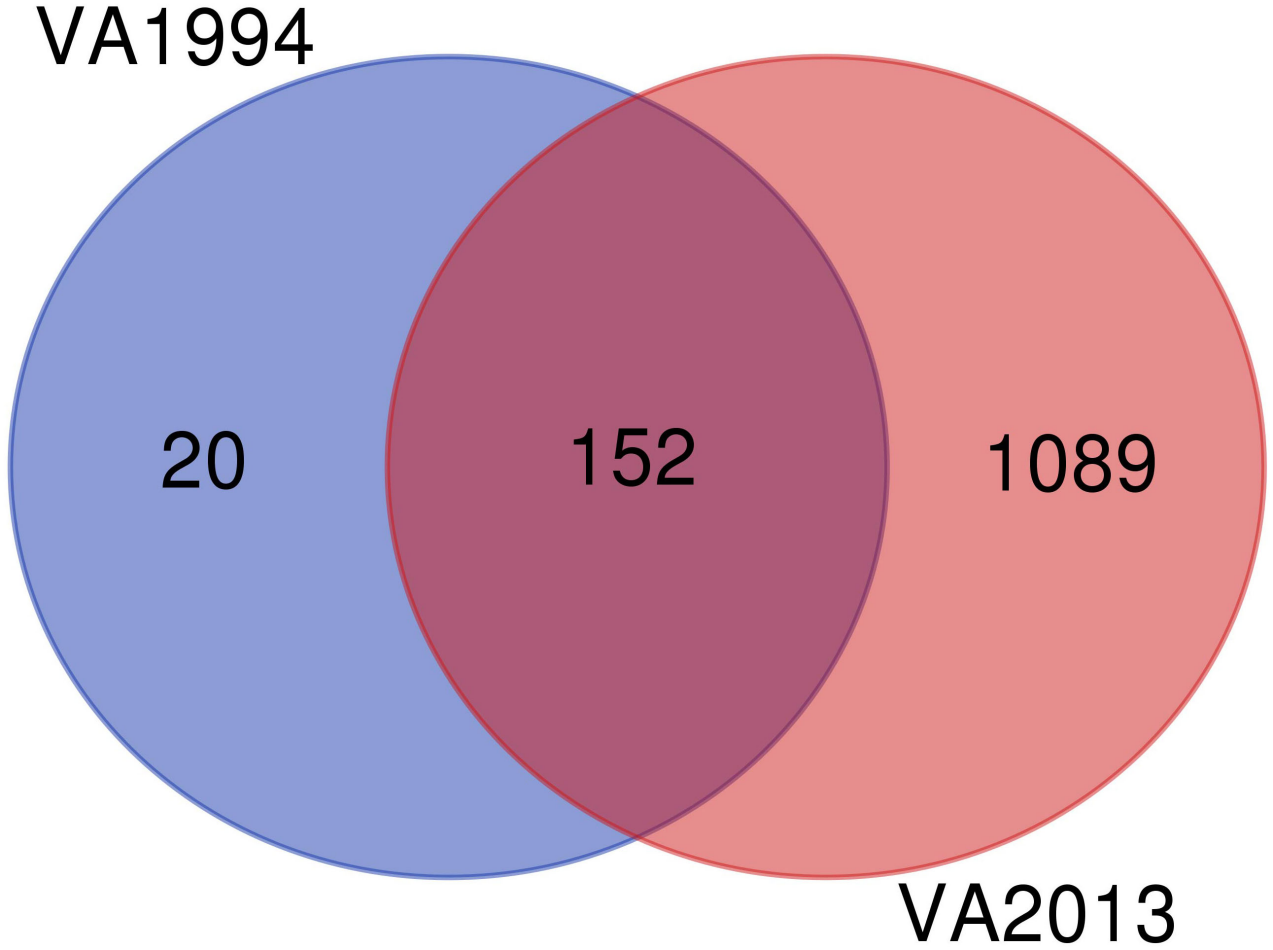
Venn diagram showing the number of differentially expressed genes (DEGs) during infection with the original *Mycoplasma gallisepticum* (MG) isolate VA1994 and the evolved isolate VA2013.

Since the DEGs common to infections with both isolates are consistent with those already discussed in the first analysis (model 1), here we focus only on the differences between the isolates. We found 20 specific genes differentially expressed on 3 DPI after inoculation with the VA1994 isolate, out of which only two genes were related with any defined immune functions: *NFATC3* and *PTAFR*, both involved in inflammation [GO:0006954] (**Figure 5**). For VA1994, there were no genes showing any significant interaction with the populations. The up-regulated and down-regulated gene interaction network for MG isolate VA1994 is shown in **Figures S13**, **S14**, ESM2.

**Figure 6:**
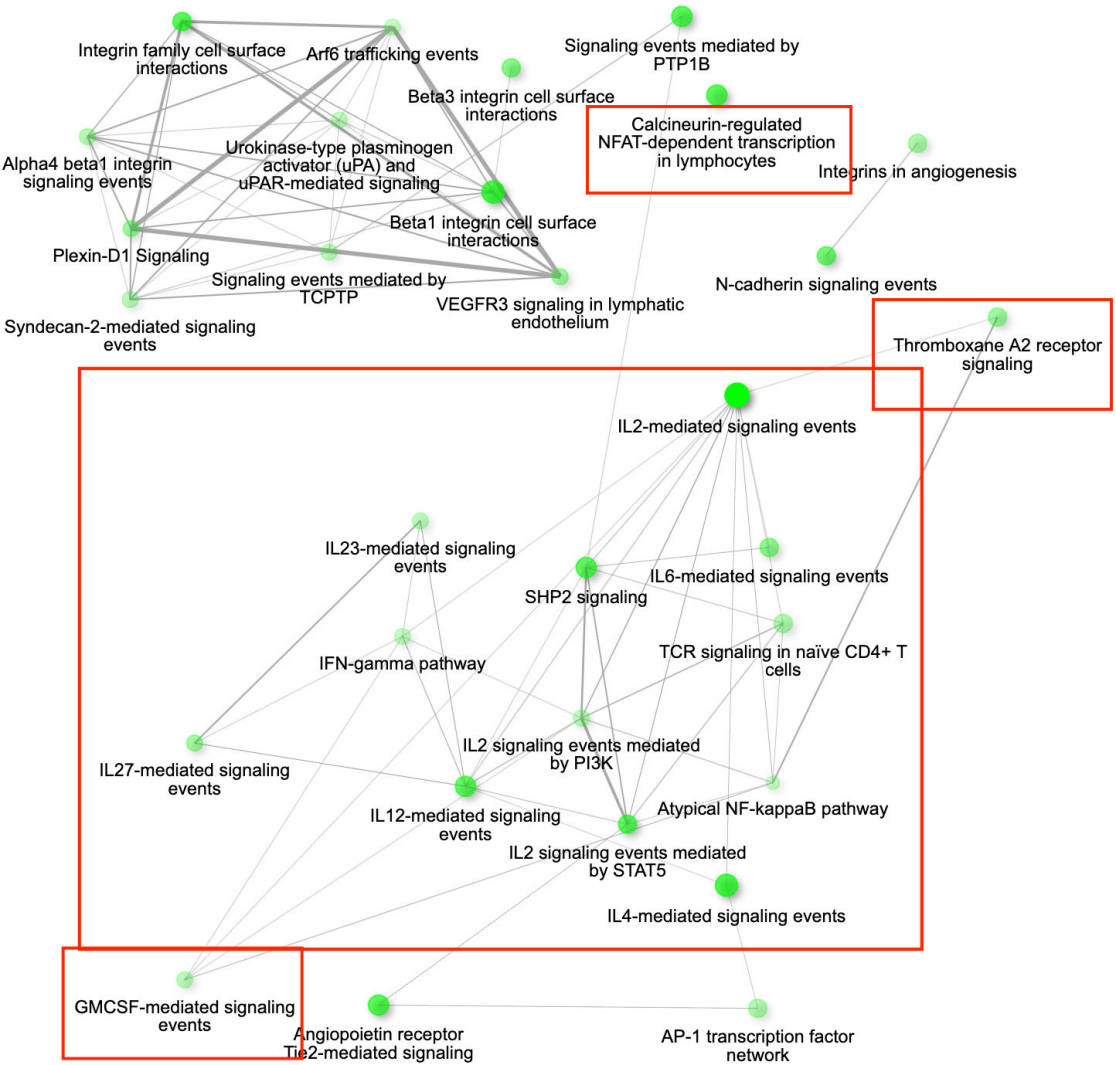
The gene interaction network for all the up-regulated differentially expressed genes (DEGs) in conjunctiva 3 days post inoculation (DPI) with *Mycoplasma gallisepticum* in birds infected with VA1994 versus VA2013. The network is showing the most significant pathways in the GO category Biological process across all the house finch populations analysed. Immune genes grouped in the pathways of our interest are highlighted in red rectangles. Node colour intensity indicates significance of gene enrichment, node size indicates number of significant DEGs.

Among the 1089 genes differentially expressed after inoculation with the MG isolate VA2013, there were 139 DEGs involved in immune function that were up-regulated, including *IL1B* (cytokine-mediated signaling pathway [GO:0019221]), *IL10* (negative regulation of cytokine activity [GO:0060302]), *IL18* (natural killer cell activation [GO:0030101)], *IL22* (inflammatory response [GO:0006954])*, TLR4* (activation of innate immune response [GO:0002218]), and *TLR7* (positive regulation of interferon-beta production [GO:0032728]) (see the pathways shown in **Figure 6**), and 39 immune DEGs that were down-regulated, including *ILRUN* (negative regulation of defense response to virus [GO:0050687])*, NTS* (positive regulation of NFKB transcription factor activity [GO:0051092])*, ROMO1* (defense response to Gram-negative bacterium [GO:0050829])*, AKAP1* (antiviral innate immune response [GO:0140374]), involved in the innate immune response, antimicrobial humoral immune response mediated by antimicrobial peptides, defense response to bacterium and antiviral innate immune response (**Supplementary Figure S15**).

Two genes were significantly differentially expressed in VA2013 in interaction with the HI population: *CNN2* had lower expression, involved in wound healing [GO:0042060] and *YWHAZ* higher expression than in VA, having role in signal transduction [GO:0007165]. There was one gene with significant interaction between the IA population and VA2013 treatment, which is a long non-coding RNA with unknown function. For the AZ population, there were two genes with significant interaction to the VA2013 treatment, again both with unknown functions.

### Differentially expressed genes commonly identified across the analyses

Finally, we searched for the genes that were identified as differentially expressed in all the three comparisons, i.e., the 1) DEGs during MG infection, 2) different pre-activation levels of expression between the populations unrelated to the MG infection, and 3) variation in expression based on the MG isolate used for the infection.

**Figure 7:**
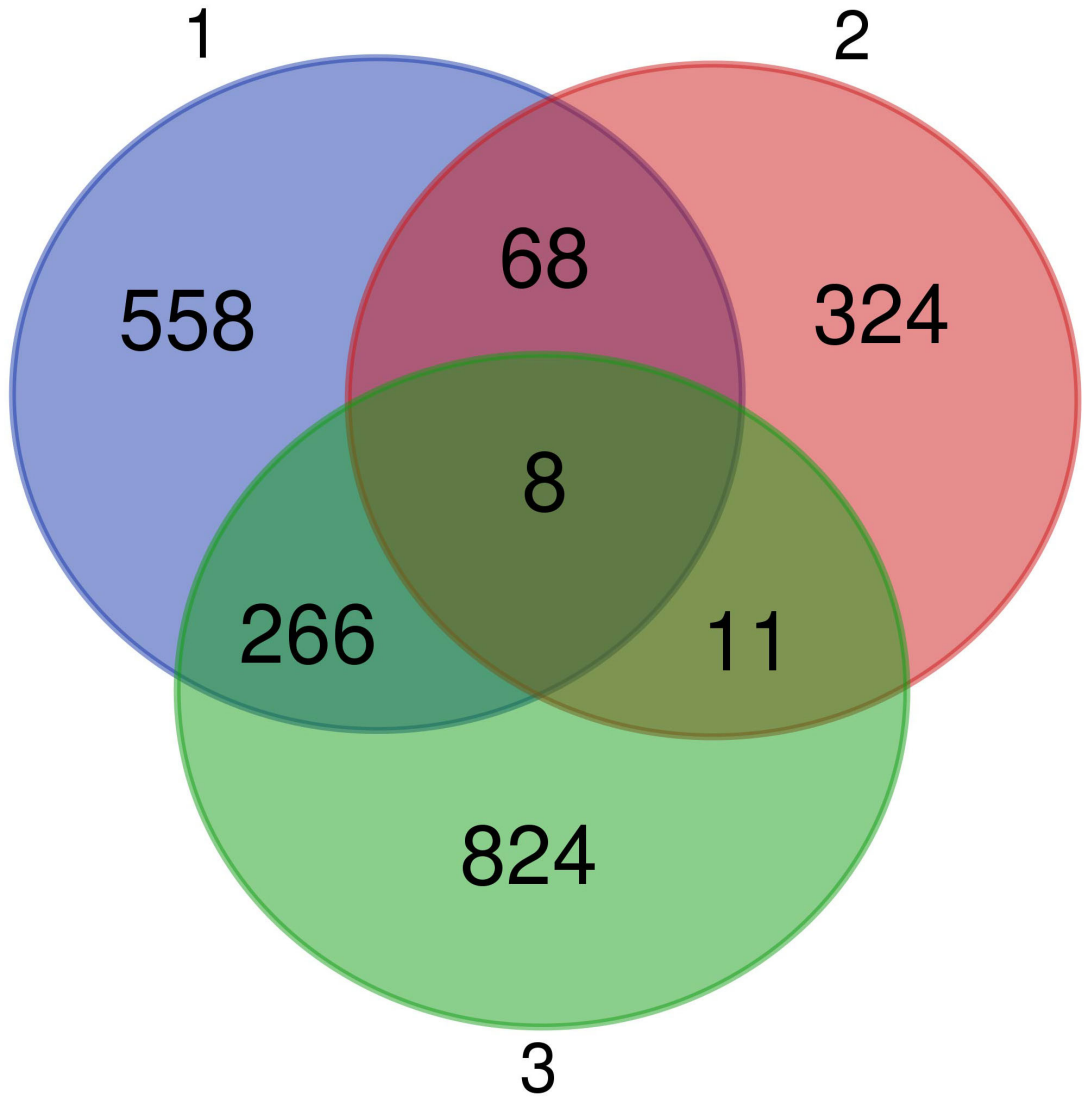
Venn diagram showing the genes in common between all the three comparative analyses performed. We found eight genes differentially expressed in conjunctiva during *Mycoplasma gallisepticum* (MG) infection (model 1), with pre-activation levels that differed among the four populations (model 2), and that differed in expression in response to the different MG isolates used for the inoculation (model 3).

We identified 8 common genes (**Figure 7**): *BCL10* integrating innate immune response [GO:0045087] and adaptive immune response regulation [GO:0002250], *USPL1* acting in cajal body organization [GO:0030576] and cell proliferation [GO:0008283], *VPS4B* acting in autophagy [GO:0016236] and cholesterol transport [GO:0030301], *RNF114* responsible for cell differentiation [GO:0030154] and protein polyubiquitination [GO:0000209], *AFMID* involved in tryptophan metabolisation to kynurenine, *ELMOD1* positively regulating the GTPase activity [GO:0019441], *CAPRIN1* responsible for negative regulation of translation [GO:0017148] and positive regulation of dendrite morphogenesis [GO:0050775] and *WDR5B* affecting histone H3-K4 methylation [GO:0051568]. Out of these genes, only BCL10 has any clear role in immunity. However, seven immune genes were also common DEGs between the first and second analysis, i.e. involved in the response to MG and also differentially pre-activated in different populations: *IL12B* regulating cellular response to IFNG [GO:0071346] and T-helper cells differentiation [GO:0042093], *PPARD* and *NR1H4* which are negative regulators of inflammatory responses [GO:0050728], including cellular responses to lipopolysaccharide [GO:0071222], *RAG1* that is key to immunoglobulin receptor recombination conditioning adaptive immune response during T-cell B-cell differentiation [GO:0002250], *RAC2* positively affecting neutrophil chemotaxis [GO:0090023] and T-cell proliferation [GO:0042129], *TRIM13* involved in positive regulation of NFKB signaling [GO:0043123] during innate immune responses, and *NCAPH2* involved in T-cell differentiation in the thymus [GO:0033077]. Finally, three immune genes showed as DEGs common to the second and third analyses, i.e. differentially pre-activated in different house finch populations and also involved in differential immune response to the two different MG isolates: *CDH17* involved in B-cell differentiation [GO:0002314], ACTG1 affecting cellular response to IFNG [GO:0071346] and *ROMO1* inducing production of reactive oxygen species (ROS) [GO:0034614], which is important in antimicrobial immune responses to bacteria.

## Discussion

Using QuantSeq 3’-end RNA transcriptomic sequencing, in this study we characterised gene expression changes in a house finch periocular lymphoid tissue, the conjunctiva, during the initial phase of infection (day 3 post inoculation) with a naturally occurring pathogen, MG. We focused on DPI 3 as a period of innate immune regulation that later guides the subsequent phases of the response either towards immunopathology-linked resistance or towards tolerance. Our focus was on the DEGs involved in the immune response and showing variation between the house finch populations differing in their co-evolutionary history with MG, as this variation may indicate adaptations of the host to MG, including in response to the increasing pathogen virulence documented previously (5). We show significant variation in expression of many inflammatory genes, especially those relevant for regulation of the Th1/Th17 pathways. In response to MG, gene expression is up-regulated at the infection site in pathogen-recognition receptors (e.g. *TLR1B*), signalling molecules and their receptors (such as *CXCL12* and *IL17R*), adaptive cell-surface receptors (*CD74*) and various other immunomodulators (e.g. *ACOD1*). Several genes important for immune response regulation varied between individuals representing house finch populations differing in their co-evolutionary history with MG (e.g., *IL12B*, *IL17*, *CASP6*, *NR1H4* or *IRF6*). Most interestingly, our data suggest that in VA, the population with the longest co-evolutionary history with MG, the birds decrease the baseline *BCL10* gene expression compared to other populations (irrespective of MG infection in model 1, and only in controls in model 2). *BCL10* also showed significant interactions between house finch populations and the MG treatment (model 1). In our analyses, *BCL10* was revealed as up-regulated during MG infection caused by the evolved VA2013 isolate (model 3). This gene has important roles in NFKB signalling and activation of both innate and adaptive immune responses, so down-regulation of its expression in the VA population may adaptively increase tolerance to infection by minimizing damaging inflammation.

Previous transcriptomic research of the house finch-MG interaction suggested that the immediate adaptation of the host to MG favoured increases in host resistance. Bonneaud et al. (39) found that house finches from populations naïve to MG experience reduced splenic immune responsiveness to MG, while the populations with a 12-year history of MG exposure (at the time of that study) have up-regulated expression of genes associated with acquired immunity in the spleen 14 days post inoculation. While this immune response can be eventually protective, allowing recovery, important costs are likely associated with such immune response. Initial results of Adelman et al. (33) indicated that in populations with longer co-evolutionary history with MG, tolerance to the infection (defined as minimizing disease severity at a given pathogen load) can contribute to improving host health. Recently, this pattern was confirmed by Henschen et al. (20), who demonstrated tolerance to MG in the eastern house finch populations with >20-year coevolutionary history with the pathogen. This study revealed that in the Harderian glands of the same birds as used in this study, up-regulated expression of some cytokines and cytokine receptors (*CXCL8*, *CXCL14*, *CCL20*, *CSF3R*) was present only in the less-tolerant populations that have not yet or only recently experienced epidemics with MG (AZ, HI). In contrast to Henschen et al. (20), our transcriptomic results in conjunctiva do not indicate clear similarities in gene expression patterns between birds from the eastern populations that share a long co-evolutionary history with MG (VA and IA), when compared to western populations (AZ and HI). This suggests that each population might have evolved a slightly different mode of regulation of the immune response to MG at the conjunctival infection site.

Our results indicate that the immune response triggered by MG 3DPI in conjunctiva represents Th17-directed inflammation. From the total 109 genes differentially expressed, the majority of immune genes (58) were up-regulated, including e.g. *TLR1B* receptor activating inflammation, IL17 receptor genes *IL17RA* and *IL17RE*, chemokine *CXCL12*, but also *ACOD1*, a negative regulator of the inflammatory response. These immune genes have significant and interspecifically conserved roles in immune activation and regulation (59–64). Similar to our results, previous transcriptomic research in chickens has also shown increases in expression of *TLR1B*, *CXCL12* and *ACOD1* after infection with MG (65–67). Some genes, such as *CD74* expressed on antigen-presenting cells (68) as a receptor for macrophage migration inhibitory factor (MIF) (69) inducing inflammation (70), showed patterns of expression contrasting with previous research in the house finch-MG system. While our data show up-regulation, Bonneaud et al. (38) reported down-regulation of *CD74* during infection. This contrast could result from the difference in tissue used, the time of tissue collection post-infection, or differences in host population coevolutionary time with MG when the studies were performed: the population with noted resistance in Bonneaud et al. (38) had ~12 years of co-evolution with MG versus 20-25 years of MG coevolution for the IA and VA populations used in this study. Increased CD74 expression during MG infection could improve activation of antigen-presenting cells (68), and through interaction with MIF (70), could also promote regenerative pathways in the tissue preventing the host damage. Overall, this could contribute to the observed host tolerance to MG in certain house finch populations. We found that only 11 immune genes were down-regulated in conjunctival tissue in response to MG, including *IL12B*, an essential mediator of the Th1 immune response. This is consistent with observations by Bonneaud et al. (39), suggesting that MG may be manipulating house finch gene expression during the acute immune response in order to allow efficient infection establishment. MG was revealed to cause immune suppression in the initial infection stages in chickens, suppressing expression of key cytokines involved in inflammation, including IL8, IL12 and CCL20 (71). Thus, our data support this hypothesis, indicating that MG may be down-regulating specific host immune pathways rather than overall immune activation.

Contrary to our expectations and to results from Harderian gland transcriptomes in the same birds (20), our general analysis of the conjunctival transcriptomes (model 1) suggested only limited interactions between MG infection status and population of origin. This result indicates tissue-specific differences in the immune regulation, but also that variation in the responses between populations may depend only on few key modifiers of the immune regulation rather than extensive transcriptome alterations. The most promising immune-controlling gene revealed in our results is *BCL10*, a positive regulator of cytokine expression involved in modulation of adaptive immune responses. In mammals, BCL10 has a vital role in channelling adaptive and innate immune signals downstream to CARMA/caspase-recruitment domain (CARD) scaffold proteins (72). BCL10 oligomerization via the CARD facilitates NFKB activation (73–75). Previous research in mice showed that BCL10 is a positive regulator of lymphocyte proliferation inducing antigen receptor signalling in B and T cells in response to NFKB activation (76). Impairment in BCL10 function negatively affects the development of memory B, CD4^+^ and CD8^+^ T cells (77). The immunomodulatory effects of *BCL10* are further documented by the up-regulation of its expression during experimental bacterial infections in cattle (78) and poultry (79). However, it has to be noted that there are also additional non-immune functions of *BCL10* described in other cells, including its involvement in neuronal regulation (80). Based on our data the precise role of BCL10 in the conjunctival tissue and causality of the changes in its expression cannot be inferred.

Although we did not find strong evidence for population differences in response to infection treatment, our results showed high number of immune genes that vary in their conjunctival expression between the house finch populations, independently of MG infection. These include key Th17 pathway regulators, such as the cytokine *IL17D* that is known to induce expression of other pro-inflammatory cytokines, including IL6 and CXCL8. This may suggest population-specific adaptations in conjunctival gene expression, potentially contributing to optimisation of the immune interaction with MG at the infection site. IL17 has a vital role in the initiation of chemotaxis and the functioning of Th17 cells (81, 82) and commonly shows up-regulation in birds immunized with various intracellular pathogens (83). Conjunctiva is colonised by innate lymphoid cells (ILCs), NK cells, γδT cells (84), αβT cells (85) and memory T cells (86), out of which the γδT cells were identified as the predominant source of IL17 during inflammation (87). In our study, *IL17D* was generally highly expressed in the AZ population, which, together with increased *BCL10*, *CASP6* and decreased *NR1H4* [a negative regulator of IL1B production; (88)] compared to the VA birds suggests disposition of the birds to resistance-oriented response through Th17 pathway pre-activation. Although the activity of *NR1H4* in conjunctiva is presently not entirely clear, its function at the site may be relevant, as in the gut this receptor negatively controls expression of a number of genes that activate inflammatory responses (58, 89, 90). In contrast to other populations, longer co-evolutionary history with MG may have selected the VA population to increase *NR1H4* and decrease *BCL10* expression, which is in agreement with the tolerance evolution described in house finches by Henschen et al. (20). This view is partially supported also by our target-gene analysis focusing on selected key immune genes with regulatory roles in immunity. All populations up-regulated *IL1B, IL6, IL10, IL18, IL22, CXCL8, CCL4, TLR1, ACOD1, TLR4*, and *TLR7* when infected with evolved MG (VA2013), which would propagate inflammation and facilitate pathogen transmission through pathological mycoplasmal conjunctivitis (15, 36). However, the AZ birds, compared to VA birds, showed a particularly high increase in expression of *TLR1* and *TLR4*, probably intensifying the resistance-oriented inflammatory response to MG. Our result thus shows similarity to the findings of Adelman et al. (33) in which house finches from populations with a longer coevolutionary history with MG (VA) showed lower inflammatory signalling and increased tolerance to infection than birds from populations with recent contact history (AZ) with MG. Further research is, however, needed to confirm the putative tolerogenic adaptations in the VA population.

Bonneaud et al. (39) proposed that the variation between house finch populations in resistance to MG likely results from some adaptations changing the initial innate immune regulation directing the subsequent adaptive immune response. This idea is consistent with the evidence from laboratory rodents showing that the initial innate immune regulation defines the efficiency of the clearance of mycoplasmal infections (91). Given the results we obtained from our general analysis (model 1), we tested this hypothesis using a subset of the data representing only the control individuals from the four house finch populations (model 2). From the high number of genes differentially expressed in the controls between the populations, 71 genes had clear roles in immunity. Consistent with our previous result, the control birds from the AZ population showed higher baseline expression of *IL17D, IL17C, IRF6, TLR15* and *TLR1B* genes putatively strengthening the overall Th17 responses, while the VA population showed stronger expression of *IL7*, *IL12B* and *LIF*, suggesting possible pre-activated Th1 immune pathway coupled with anti-inflammatory signalling, which was again linked with decreased *BCL10* expression. We assume that immunological regulation of tolerance to infection must involve balanced changes of both pro- and anti- inflammatory pathways to prevent infection-caused mortality. *IL12B*, a subunit of IL12, primarily stimulates natural killer (NK) cells and induces the differentiation of naive CD4^+^ T lymphocytes into T helper 1 (Th1) effectors (92). If the IL12B subunit is dimerized with the IL23A subunit, then functional IL23 is produced (93), which is necessary for Th17 development and function (94). Alternatively, *IL12B* can also mediate anti-inflammatory regulation increasing expression of other regulatory cytokines such as IL10 (95), with IL7 supporting the host defence by regulating immune cell growth and homeostasis (96). Thus, increased baseline expression of *IL12B* might have multiple functional roles in protecting the health of the VA birds during the onset of MG infection. Birds from the HI and IA populations showed similar up-regulation of immune-related pathways activated by mast cells and B cells (*TRIM13* and *PPARD*) when compared with the VA birds but also with AZ birds. Taken altogether, the pattern of immune gene expression in the VA birds was different from all the other three remaining house finch populations, putatively resulting, at least in part, from long-lasting adaptation to MG through a combination of resistance and tolerance (20).

We also examined pathogen contributions to differential conjunctival gene expression across populations (model 3). Consistent with previous research (5, 20, 37) we found that the evolved (VA2013) isolate triggers much stronger conjunctival immune responses than the original (VA1994) one, here indicated by the number of DEGs when compared to controls. In contrast to VA1994, the evolved isolate VA2013 activated pathways involving differential expression of both pro-inflammatory and anti-inflammatory genes, including key signal mediators such as *IL1B*, *IL10*, *IL18*, *IL22* and *CXCL8*. Especially negative regulators of inflammation, such as IL10, can play important roles in fine-tuning immunomodulation, since their down-regulation can improve pathogen clearance, but also increase tissue damage (97–100), optimising the immunity-immunopathology balance in the defence (9). Previous research in rodents performed both *in vivo* and *in vitro* shows that *Mycoplasma pneumoniae* antigens induce potent immune reactions through enhancement of the Th17 response, but regulatory T cell (Treg) activation linked with *IL10* expression simultaneously suppress *IL17A* expression (101). In contrast, *IL18* is a potent pro-inflammatory cytokine regulating both innate and acquired immune responses (102). Studies in chicken show that MG infection increased mRNA levels of *IL18* between 3 and 7 DPI, similar to our results (103). Also IL22 is a key mediator of inflammation that is produced immediately after stimulation to initiate an immune response, mediating also mucous production, wound healing, and tissue regeneration (104). Comparable to our results, *IL22* gene has been reported as up-regulated during *Mycoplasma ovipneumoniae* infection in sheep (105).

Overall, comparison of the results from all three analyses performed identifies *BCL10* as a potentially important immune gene that changes its conjunctival expression during the MG infection, varies in its expression between individuals from different house finch populations, and also varies in expression depending on the MG isolate infecting the birds. Furthermore, other genes involved in the response to MG (model 1 or model 3) and at the same time also differentially pre-activated in distinct host populations (model 2) may be of high importance for house finch adaptation to MG. Our results elucidated both positive and negative regulators of inflammation and Th1 immunity, including *IL12B* and possibly also *PPARD* and *NR1H4*. Roles of other genes repeatedly revealed in our analyses are less clear, but they may contribute to altered leukocyte differentiation, infiltration into the tissue or cell activation (*RAG1, RAC2, TRIM13, NCAPH2, CDH17, ACTG1* and *ROMO1*). Thus, all these 11 genes potentially provide adaptations to the selective pressures posed by MG varying between the house finch populations.

Our transcriptomic results obtained in conjunctiva apparently differ from the results obtained earlier by Henschen et al. (20) from the same experiment but for a different tissue, the Harderian gland. Most importantly, the pattern of variation between the house finch populations revealed for the two tissues in response to MG is different. While we assume that biologically significant differences in immune regulation between the tissues are responsible for the differences in gene expression patterns observed, we are, unfortunately, presently unable to explain them, because for the two studies different transcriptomic methods were adopted, RNA-seq and QuantSeq, respectively. The RNA-seq approach can be biased by more enriched DEGs for longer transcripts than for the shorter ones (106). Previous research has reported that RNA-seq identifies in general more DEGs, but QuantSeq can detect more of the shorter transcripts (46) that often act in immunity (107). Thus, future research is needed to validate the results and reveal if the difference in the transcriptomic results obtained for the two house finch tissues reflect true biological difference between the tissues, variation in the transcriptomic approaches adopted, or both.

## Conclusion

Our results illuminate potential immunological pathways underlying increased tolerance to MG in birds from the VA population compared to the other house finch populations. Notably, they suggest the importance of evolving balance between the Th1 and Th17 pathway activation during the initial conjunctival response of the house finches to the MG infection. The populations in no or only recent contact with MG may have increased tendency for up-regulation of the *IL17*-linked pathway (observed in AZ), while the populations with long-established co-evolutionary history with MG (VA), could promote *IL12* signalling to increase Th1 and/or anti-inflammatory (possibly B-cell driven) immune responses. Further research should focus on understanding of specific roles of various cell types in the immune responses to MG in birds from populations differing in their co-evolutionary history with MG. Furthermore, our results also document that infection with a more recent MG isolate (VA2013) triggers in conjunctiva stronger expression of immune genes than infection with the original isolate (VA1994). Since also non-immune pathways may be affected by this regulation [e.g. pathways regulating the extent of the sickness behaviour which might influence MG transmission in the finches; (36, 108)], further research should also investigate the expression changes in genes with other functions expressed in non-lymphoid tissues.

## Data availability statement

The data presented in the study are deposited in the NCBI BioProject repository, accession number PRJNA981079.

## Ethics statement

The animal study was approved by Institutional Animal Care and Use Committees (IACUC) at Iowa State University (ISU) and Virginia Tech, and the ISU Institutional Biosafety Committee. The study was conducted in accordance with the local legislation and institutional requirements.

## Author contributions

Conceptualization: NKV, AEH, RAD, DMH, JSA, MV. Data Curation: NKV, AEH, RAD, DMH, JSA. Formal Analysis: NKV, BM. Funding Acquisition: NKV, BM, RAD, DMH, JSA, MV. Investigation: NKV, AEH, BM, RAD, DMH, JSA, MV. Methodology: NKV, AEH, BM, VB, RAD, DMH, JSA, MV. Project Administration: NKV, BM, AEH, DMH, JSA, MV. Resources: MV, RAD, DMH, JSA. Software: n/a. Supervision: AEH, RAD, DMH, JSA, MV. Validation: NKV, AEH, RAD, DMH, JSA, MV. Visualization: NKV. Writing – Original Draft Preparation: NKV, MV. Writing – Review and Editing: NKV, AEH, BM, RAD, VB, DMH, JSA, MV. All authors contributed to the article and approved the submitted version.
