# Supplementary material for: Varying conjunctival immune response adaptations of house finch populations to a rapidly evolving bacterial pathogen
Source: Front Immunol. 2024 Feb 2;15:1250818. doi: 10.3389/fimmu.2024.1250818 (PMC10869556; doi:10.3389/fimmu.2024.1250818)
Supplement: Supplementary file 1 [file DataSheet_1.pdf]

Electronic Supplementary Material 2

Supplementary Figures

Title: Varying conjunctival immune response adaptations of house finch populations to a rapidly evolving bacterial pathogen

Authors: Nithya Kuttiyarthu Veetil, Amberleigh E. Henschen, Dana M. Hawley , Balraj Melepat, Rami A. Dalloul, Vladimír Beneš, James S. Adelman and Michal Vinkler

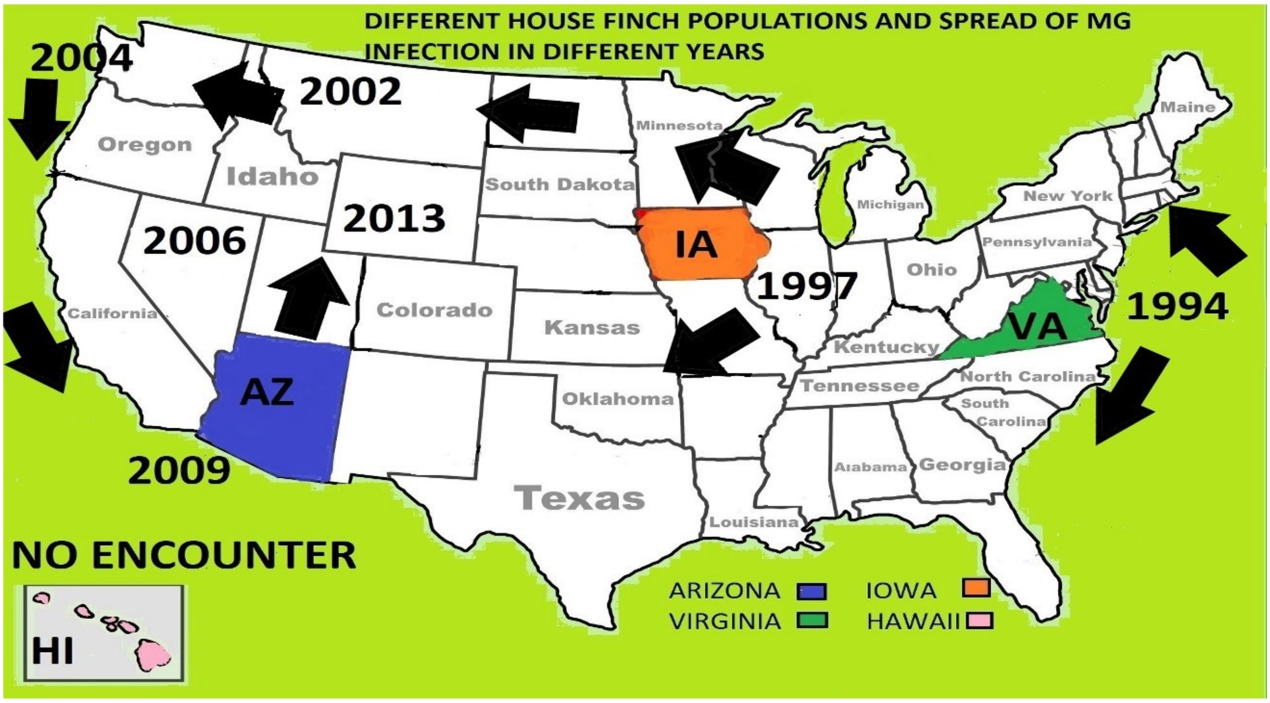

Figure S1. A map delineating the infection of Mycoplasma gallisepticum within house finches is presented, illustrating the temporal development of the disease. The locations where bird collections were conducted—specifically, Virginia (VA), Iowa (IA), Arizona (AZ), and Hawaii (HI)—have been distinctly highlighted on the map.

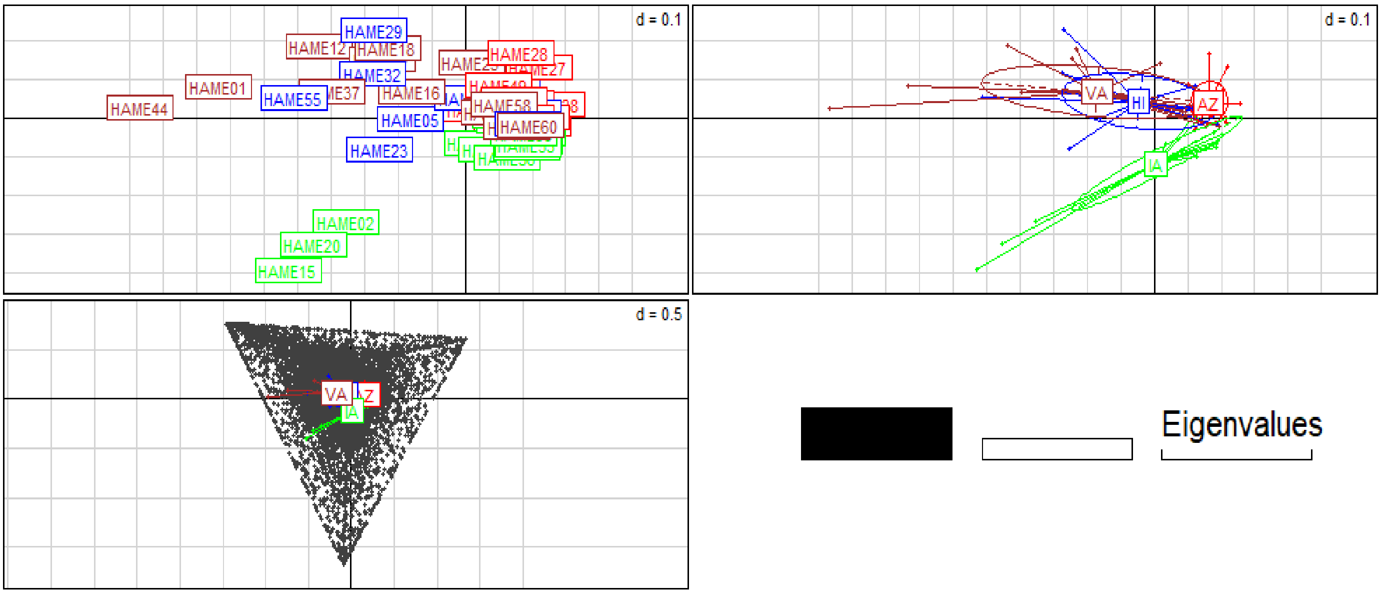

Figure S2. Between group analysis (BGA) results of transcriptomic data where individual populations served as the grouping factor. VA = Virginia (dark red), IA = Iowa (green), AZ = Arizona (red) and HI = Hawaii (blue)

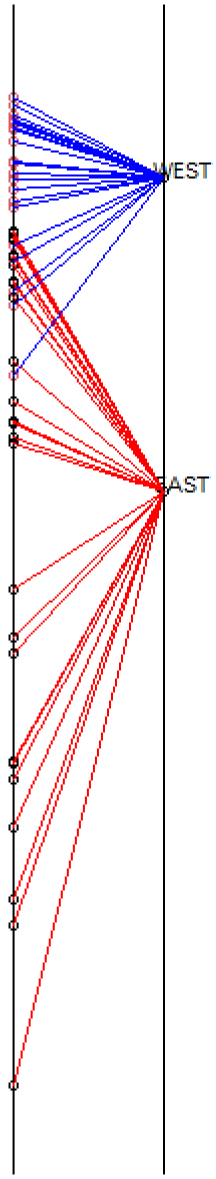

Figure S3. Between group analysis (BGA) results of transcriptomic data where the transcriptomic dataset was divided between east (VA and IA) and west (AZ and HI) populations differing in co-evolutionary history with MG.

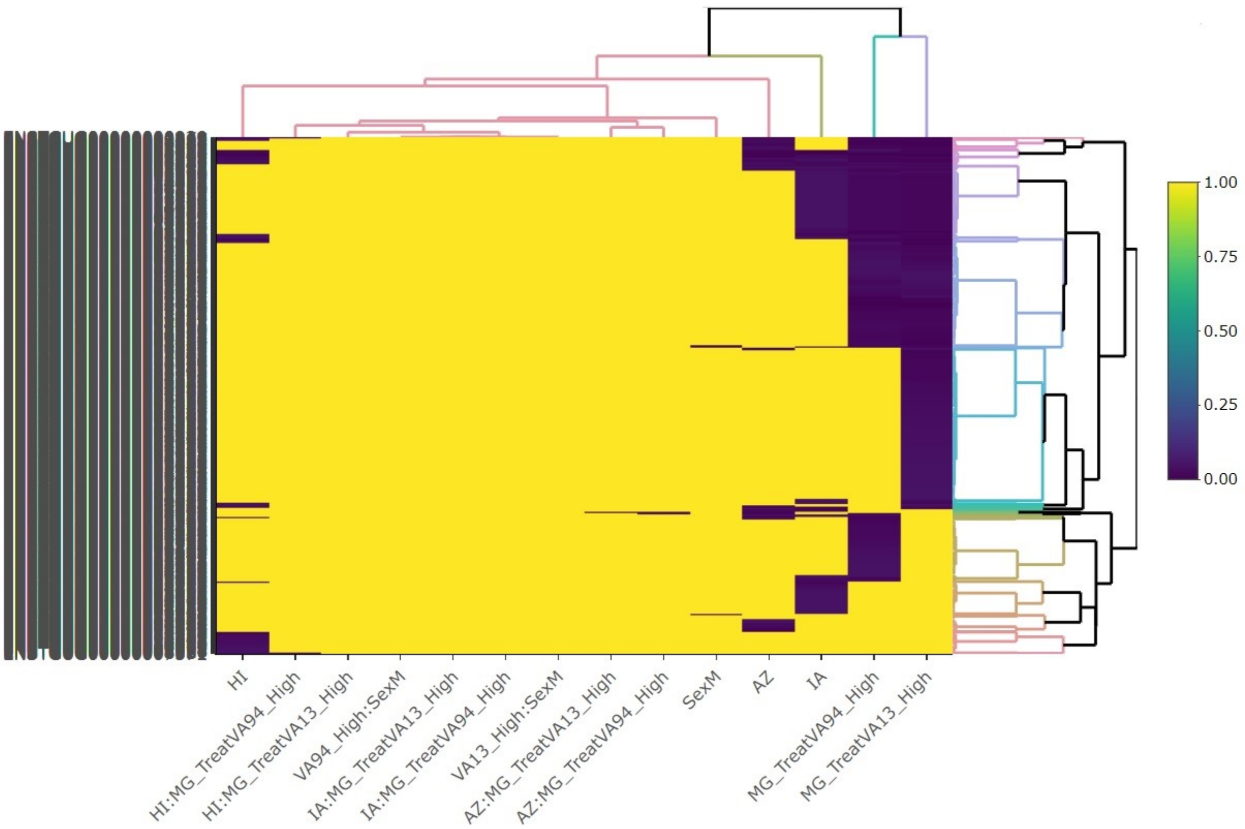

Figure S4. Heatmap showing the differential gene expression across the investigated house finch populations with respect to *Mycoplasma gallisepticum* (MG) treatment and sex, with interactions. Statistical significance indicated with FDR corrected p value ranging from 0.00 to 1.00.

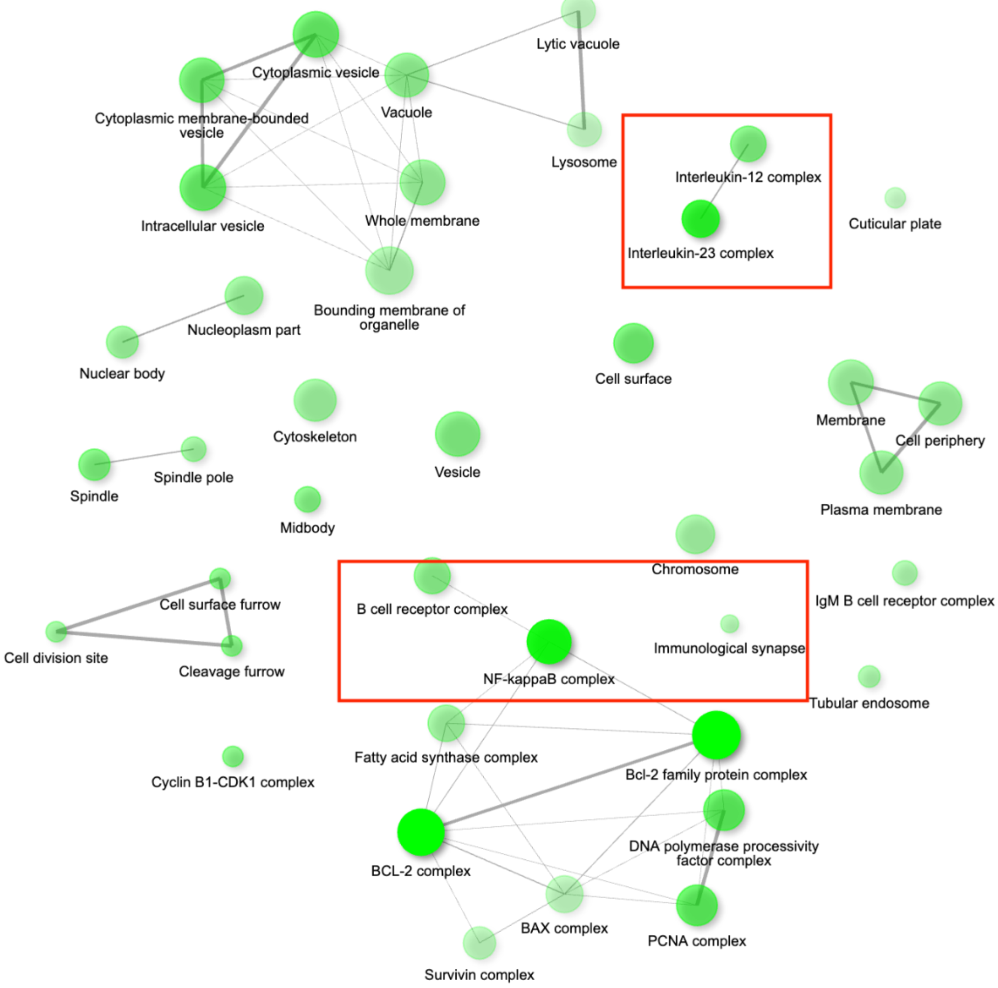

Figure S5. The gene interaction network for the differentially expressed genes (DEGs) down-regulated during the MG infection showing the most significant pathways in the GO category biological processes with clear immune function. Node colour intensity indicates significance of gene enrichment, node size indicates number of significant DEGs

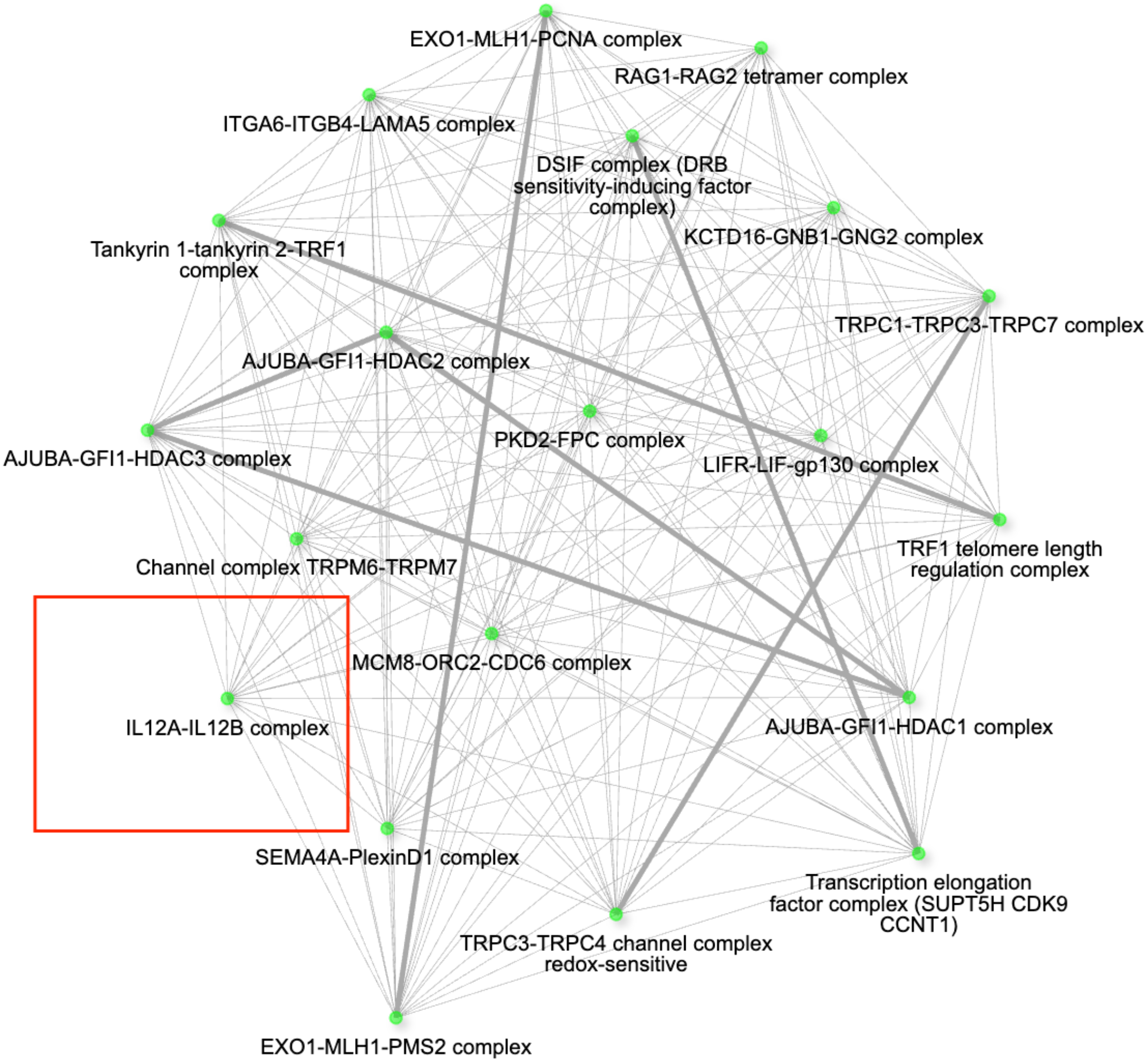

Figure S6. The gene interaction network for the differentially expressed genes (DEGs) down-regulated in the control bird populations of Iowa (IA), Arizona (AZ) and Hawaii (HI) compared to Virginia (VA) birds showing the most significant pathways in the GO category Biological process**.** Immune genes grouped in the pathways of our interest are highlighted with red rectangles. Node colour intensity indicates significance of gene enrichment, node size indicates number of significant DEGs.)

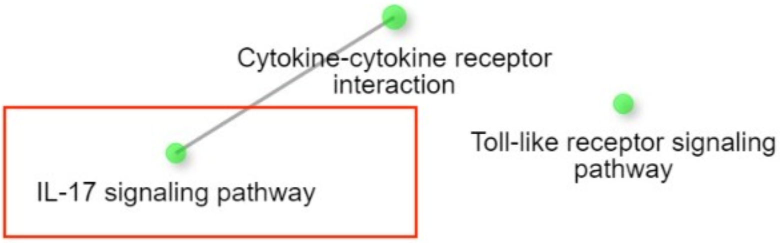

Figure S7. The gene interaction network for the differentially expressed immune genes (DEGs) up-regulated in the control bird populations of Arizona (AZ) compared to

Virginia (VA) birds showing the most significant pathways in the GO category Biological process**.** Immune genes grouped in the pathways of our interest are highlighted with red rectangles. Node colour intensity indicates significance of gene enrichment, node size indicates number of significant DEGs.)

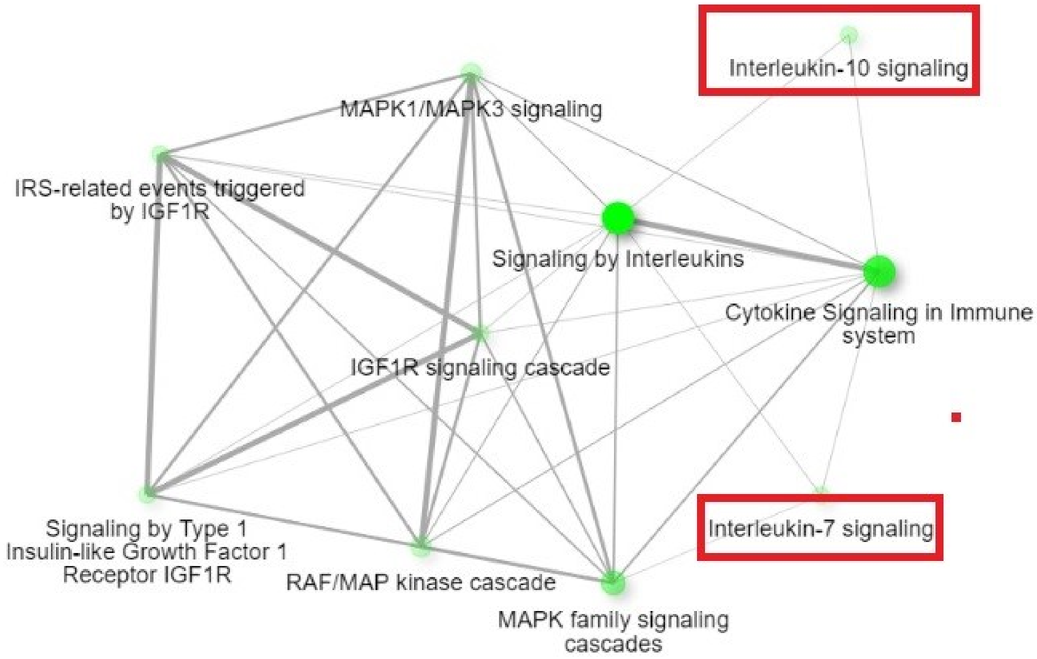

Figure S8. The gene interaction network for the differentially expressed immune genes (DEGs) down-regulated in the control bird populations of Arizona (AZ) compared to Virginia (VA) birds showing the most significant pathways in the GO category Biological process**.** Immune genes grouped in the pathways of our interest are highlighted with red rectangles. Node colour intensity indicates significance of gene enrichment, node size indicates number of significant DEGs.)

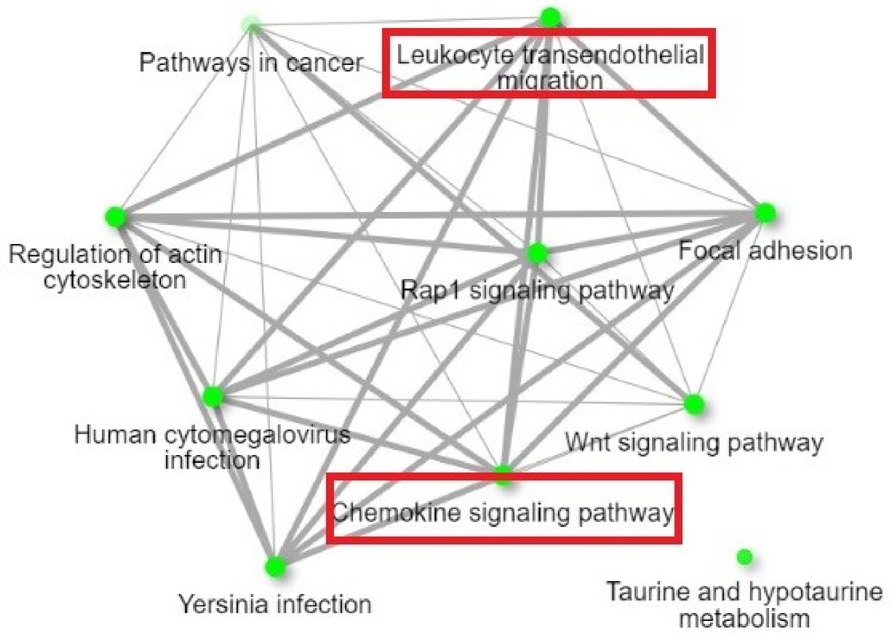

Figure S9. The gene interaction network for the differentially expressed immune genes (DEGs) up-regulated in the control bird populations of Hawaii (HI) compared to Virginia (VA) birds showing the most significant pathways in the GO category Biological process**.** Immune genes grouped in the pathways of our interest are highlighted with red rectangles. Node colour intensity indicates significance of gene enrichment, node size indicates number of significant DEGs.)

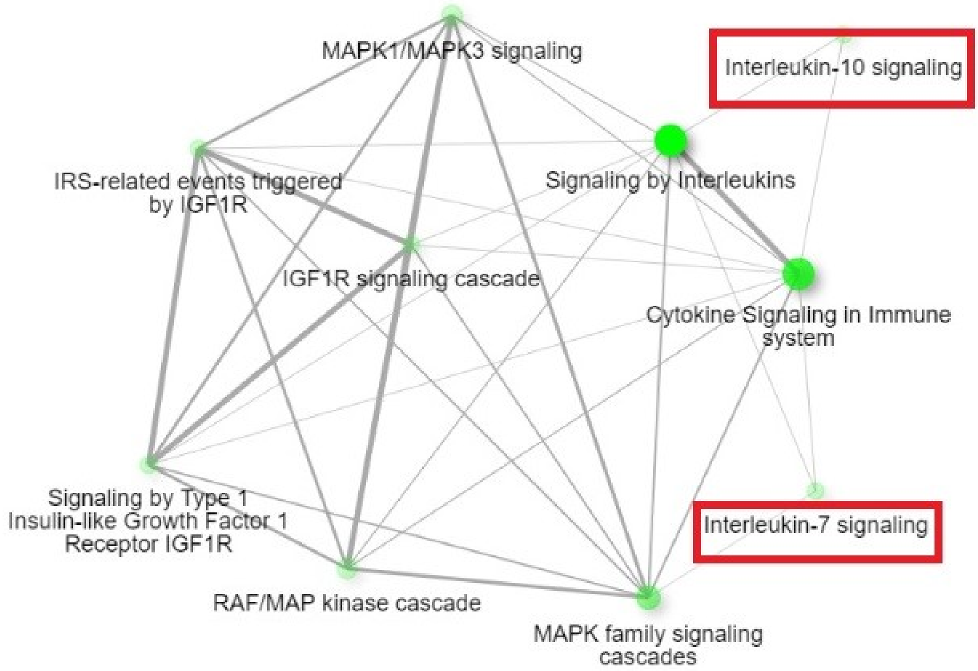

Figure S10. The gene interaction network for the differentially expressed immune genes (DEGs) down-regulated in the control bird populations of Hawaii (HI) compared to Virginia (VA) birds showing the most significant pathways in the GO category Biological process**.** Immune genes grouped in the pathways of our interest are

highlighted with red rectangles. Node colour intensity indicates significance of gene enrichment, node size indicates number of significant DEGs.)

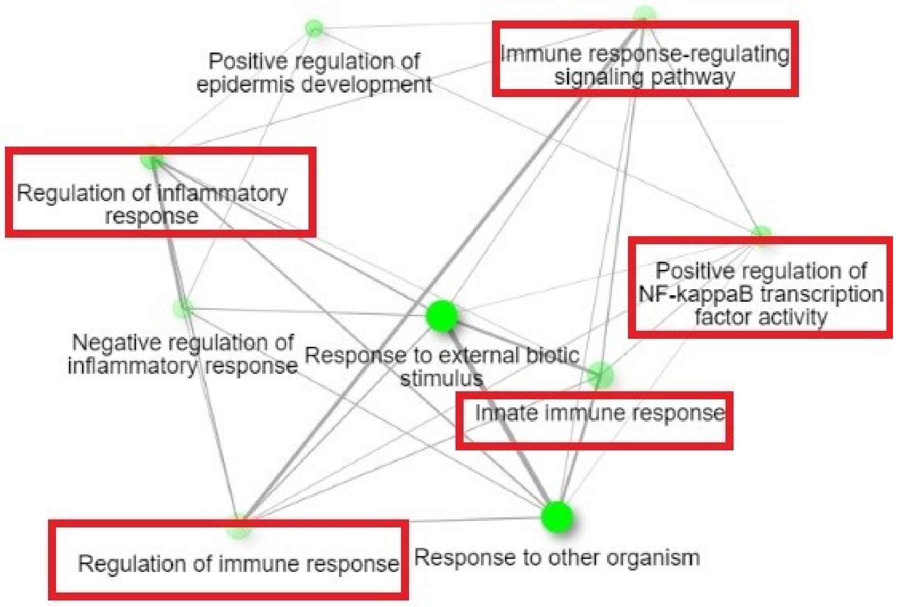

Figure S11. The gene interaction network for the differentially expressed immune genes (DEGs) up-regulated in the control bird populations of Iowa(IA) compared to Virginia (VA) birds showing the most significant pathways in the GO category Biological process**.** Immune genes grouped in the pathways of our interest are highlighted with red rectangles. Node colour intensity indicates significance of gene enrichment, node size indicates number of significant DEGs.)

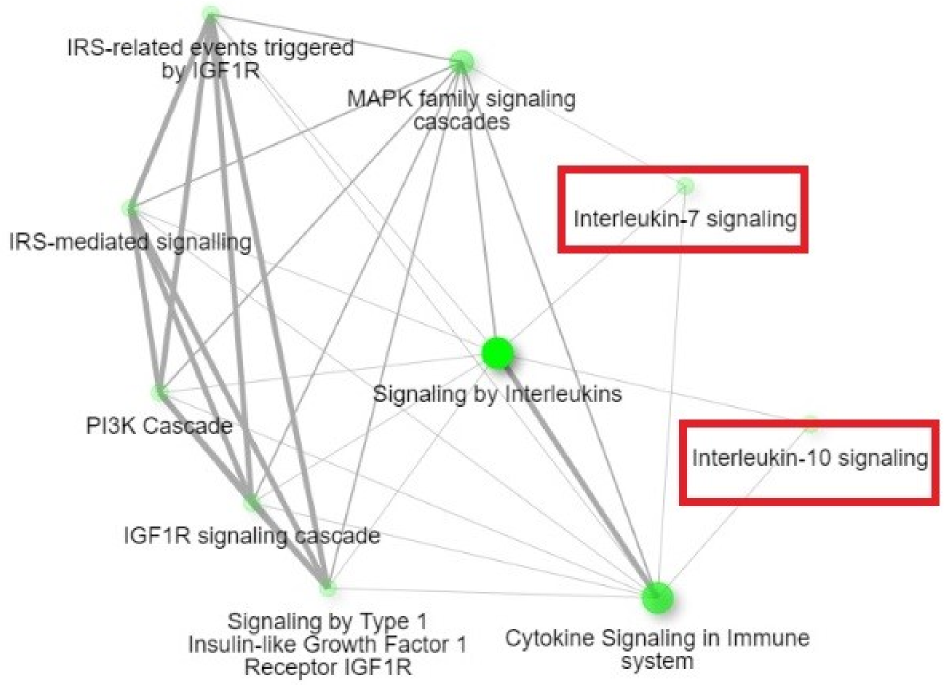

Figure S12. The gene interaction network for the differentially expressed immune genes (DEGs) down-regulated in the control bird populations of Iowa (IA) compared to Virginia (VA) birds showing the most significant pathways in the GO category Biological process**.** Immune genes grouped in the pathways of our interest are highlighted with red rectangles. Node colour intensity indicates significance of gene enrichment, node size indicates number of significant DEGs.)

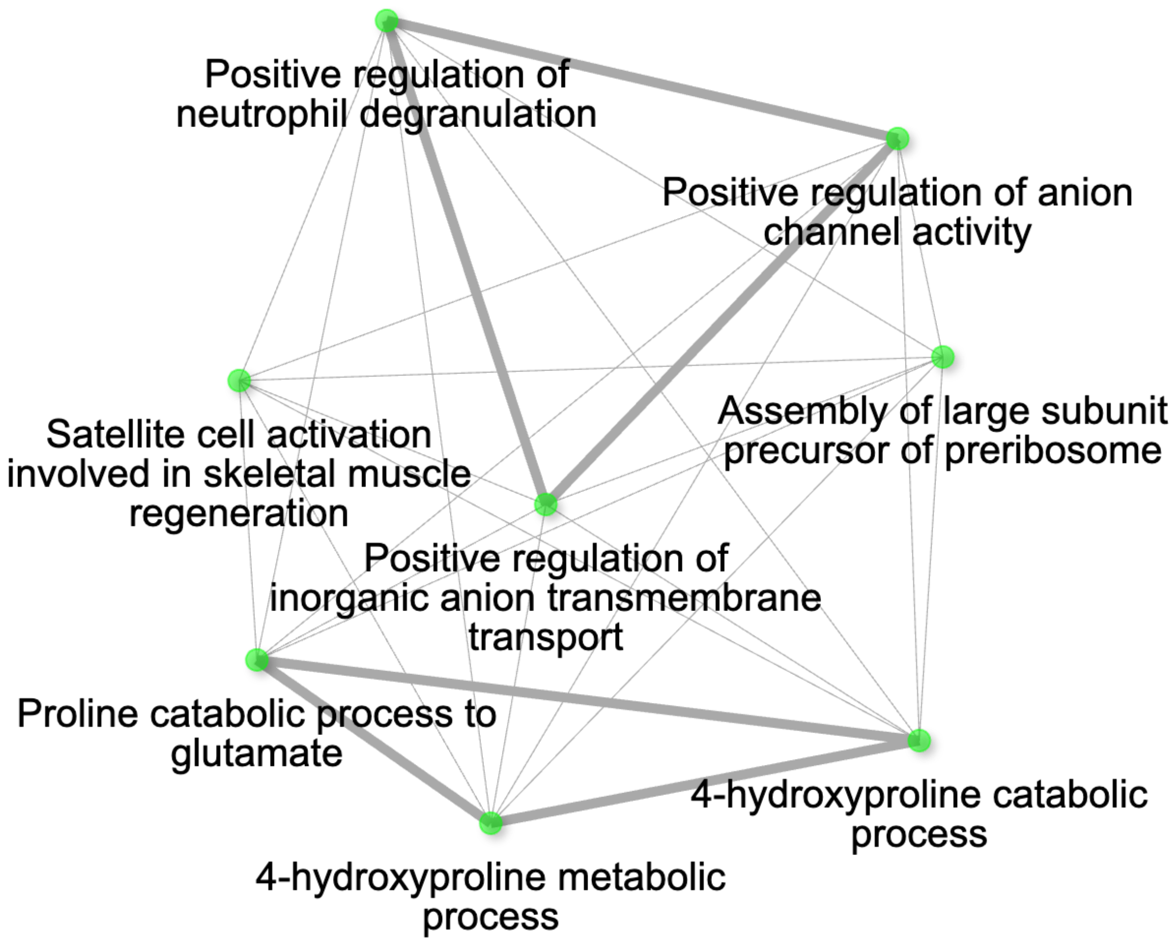

Figure S13. The gene interaction network for all the up-regulated DEGs in VA1994 different from those identified for VA2013 showing the most significant pathways in the GO category biological process. Immune genes grouped in the pathways of our interest are highlighted in red rectangles. Immune genes grouped in the pathways of our interest are highlighted with red rectangles. Node colour intensity indicates significance of gene enrichment, node size indicates number of significant DEGs.)

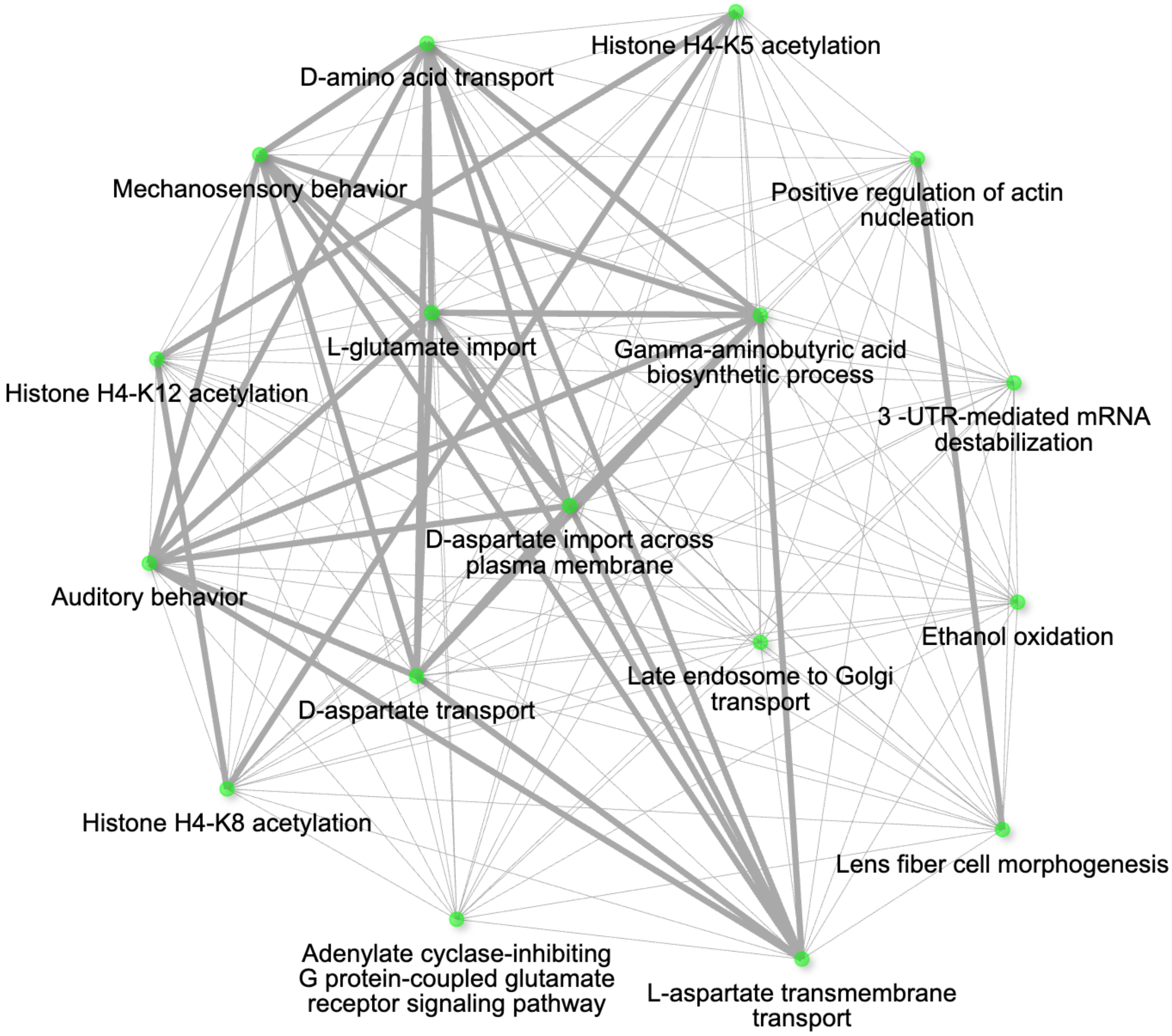

Figure S14. The gene interaction network for all the down-regulated DEGs in VA1994 different from those identified for VA2013 showing the most significant pathways in the GO category biological process. Immune genes grouped in the pathways of our interest are highlighted in red rectangles. Immune genes grouped in the pathways of our interest are highlighted with red rectangles. Node colour intensity indicates significance of gene enrichment, node size indicates number of significant DEGs.)

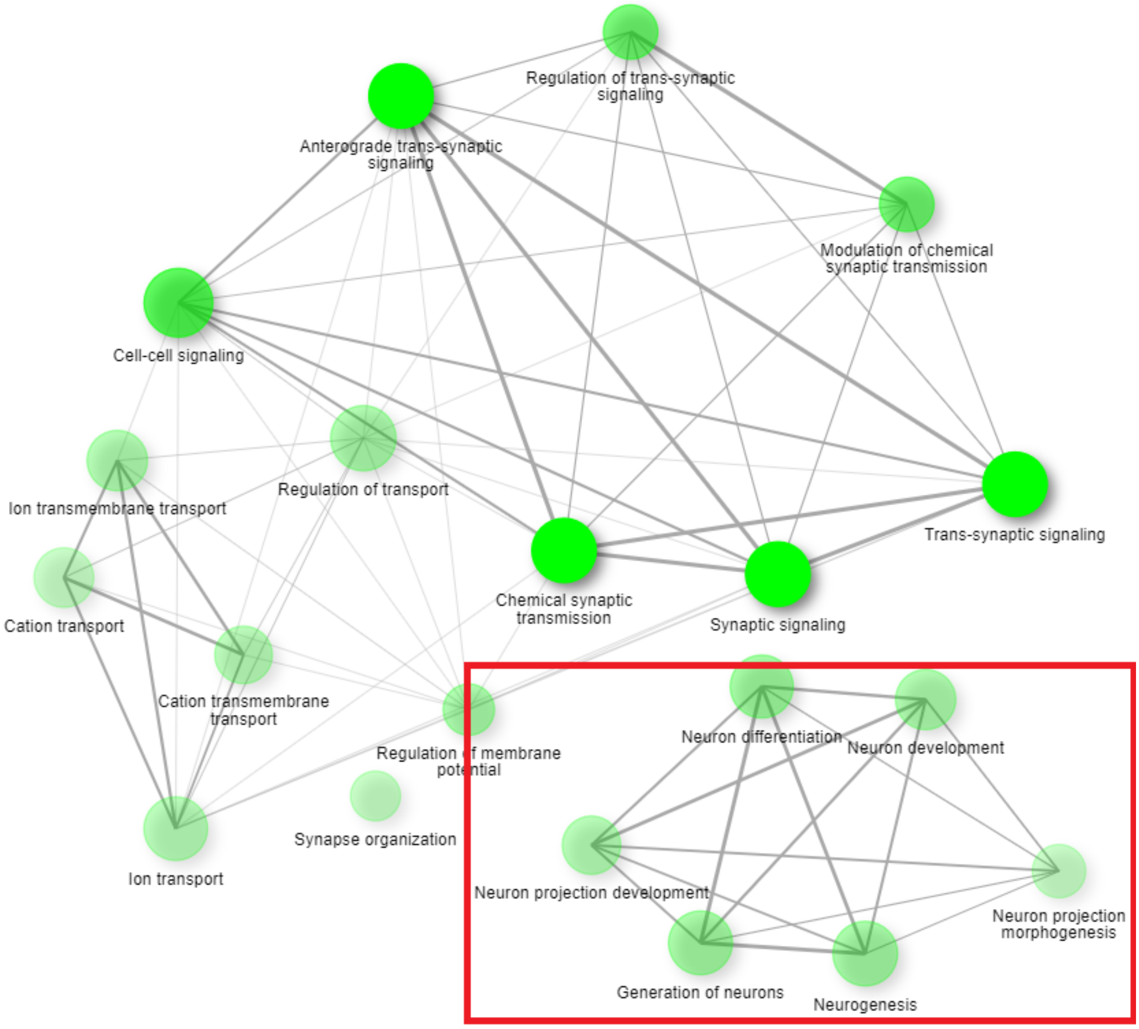

Figure S15. The gene interaction network for all the down-regulated DEGs in VA2013 different from those identified for VA1994 showing the most significant pathways in the GO category biological process. Neurogenesis related genes grouped in the pathways of our interest are highlighted in red rectangles. Node colour intensity indicates significance of gene enrichment, node size indicates number of significant DEGs.)
